# Mesenchymal stromal cells in bone marrow niche of patients with multiple myeloma: a double-edged sword

**DOI:** 10.1186/s12935-025-03741-x

**Published:** 2025-03-26

**Authors:** Sina Kamrani, Reza Naseramini, Pouria Khani, Zahra Sadat Razavi, Hamed Afkhami, Mohammad Reza Atashzar, Farzad Nasri, Sajad Alavimanesh, Farzane Saeidi, Hossein Ronaghi

**Affiliations:** 1 Department of Orthopedic, Faculty of Medicine, Guilan University of Medical Sciences, Rasht, Iran; 2 Department of Medical Genetics, School of Medicine, Tehran University of Medical Sciences (TUMS), Tehran, Iran; 3 Physiology Research Center, Iran University of Medical Sciences, Tehran, Iran; 4 Cellular and Molecular Research Center, Qom University of Medical Sciences, Qom, Iran; 5 Nervous System Stem Cells Research Center, Semnan University of Medical Sciences, Semnan, Iran; 6 Department of Medical Microbiology, Faculty of Medicine, Shahed University, Tehran, Iran; 7 Department of Immunology, School of Medicine, Fasa University of Medical Sciences, Fasa, Iran; 8 Department of Immunology, School of Medicine, Iran University of Medical Sciences, Tehran, Iran; 9 Student Research Committee, Shahrekord University of Medical Sciences, Shahrekord, Iran; 10 Cellular and Molecular Research Center, Basic Health Sciences Institute, Shahrekord University of Medical Sciences, Shahrekord, Iran; 11 Department of Medical Genetics, School of Medical Sciences, Tarbiat Modares University, Tehran, Iran

**Keywords:** Multiple myeloma, Bone marrow mesenchymal stromal cells, Myeloma progression

## Abstract

Multiple myeloma (MM) is a hematological malignancy defined by the abnormal proliferation and accumulation of plasma cells (PC) within the bone marrow (BM). While multiple myeloma impacts the bone, it is not classified as a primary bone cancer. The bone marrow microenvironment significantly influences the progression of myeloma and its treatment response. Mesenchymal stromal cells (MSCs) in this environment engage with myeloma cells and other bone marrow components via direct contact and the secretion of soluble factors. This review examines the established roles of MSCs in multiple facets of MM pathology, encompassing their pro-inflammatory functions, contributions to tumor epigenetics, effects on immune checkpoint inhibitors (ICIs), influence on reprogramming, chemotherapy resistance, and senescence. This review investigates the role of MSCs in the development and progression of MM.

**Supplementary Information:**

The online version contains supplementary material available at 10.1186/s12935-025-03741-x.

## Introduction

**Fig. 1:**
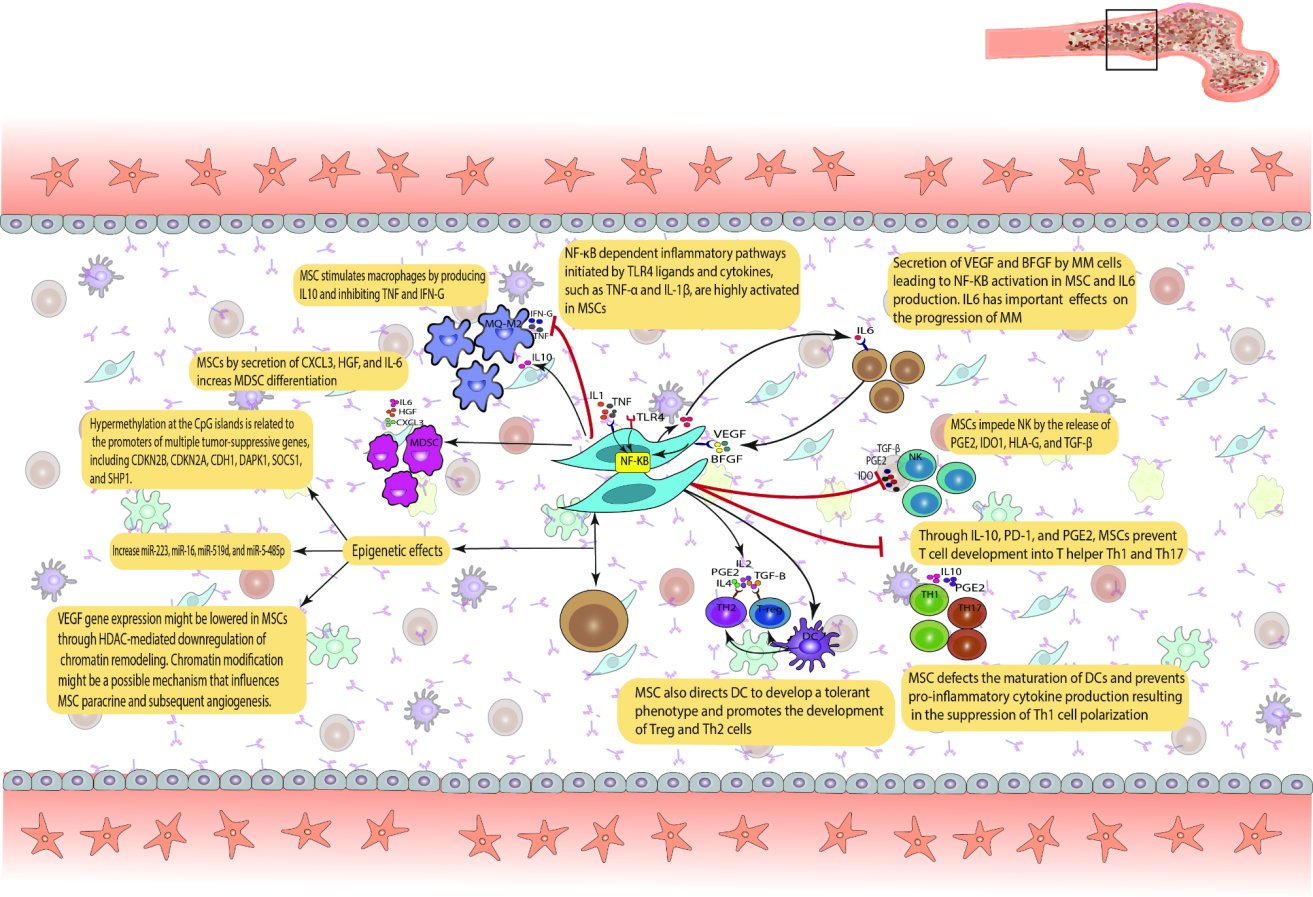
Schematic representation of the interaction between Multiple Myeloma (MM) cells and Mesenchymal Stromal Cells (MSCs) in the bone marrow niche. This figure illustrates how MSCs influence MM progression through cytokine signaling, immune modulation, angiogenesis, and chemotherapy resistance

Mesenchymal stromal cells (MSCs) located in the bone marrow (BM) niche are pivotal in the pathogenesis of multiple myeloma (MM), functioning as a dual entity that can either facilitate or impede tumour progression (Fig. 1).

MSCs play a vital role in normal hematopoiesis and tissue regeneration; however, their interaction with MM cells results in complex and occasionally contradictory effects. Mesenchymal stem cells (MSCs) play a dual role in multiple myeloma (MM) progression by facilitating immune evasion, angiogenesis, and chemoresistance, while also possessing the capacity to inhibit tumor growth under specific circumstances. The dual nature of MSCs in MM raises questions regarding their precise role in the disease and their potential as therapeutic targets [1].

The bone marrow niche in the musculoskeletal system is defined by a dynamic microenvironment that includes multiple cell types, such as mesenchymal stem cells, osteoblasts, osteoclasts, endothelial cells, immune cells, and hematopoietic cells (Table 1). The microenvironment is crucial for both the maintenance of hematopoiesis and the progression of multiple myeloma (MM), as malignant plasma cells (PCs) exploit the bone marrow (BM) to enhance their survival and proliferation. Upon infiltrating the bone marrow, multiple myeloma cells disrupt the balance between osteoblasts and osteoclasts, resulting in bone destruction and the formation of osteolytic lesions. Mesenchymal stem cells (MSCs) play a role in these alterations by facilitating osteoclast activation and supporting tumor cell survival via multiple signalling pathways [2, 3].

**Table 1:** MSC properties in healthy and multiple myeloma bone marrow microenvironments

Mesenchymal stromal cells	Normal	Multiple myeloma	References
Cell size	Small	Large	[35]
NF-KB activation	Low	High	[36]
Inflammatory cytokines such as IL-6, TNF	Low	High	[37]
T-helper cell differentiation	Th1, Th17	Th2, Treg	[38]
Macrophage phenotypes	M1 MQ	M2 MQ	[39]
Signaling pathway	WNT pathway	NOTCH pathway	[40]
Osteogenic differentiation potential	High	Low	[41]

Osteoclasts, an essential cell type in the bone marrow niche, are integral to the bone-resorbing process that is crucial in the pathogenesis of multiple myeloma (MM). Mesenchymal stem cells (MSCs) significantly influence osteoclast activation through the secretion of factors such as RANKL and M-CSF, which facilitate osteoclast differentiation and activity. In multiple myeloma, mesenchymal stem cells (MSCs) play a role in the development of osteolytic bone lesions by promoting osteoclastogenesis [4]. The reciprocal interaction between MSCs and osteoclasts establishes a cycle that intensifies bone destruction and supports the survival of myeloma cells. Additionally, osteoclast-mediated bone resorption releases factors such as TGF-β and IGF-1, which further enhance the growth and survival of MM cells. This highlights the significant function of MSCs in regulating the equilibrium between bone formation and resorption in the BM microenvironment, an equilibrium that, when altered, promotes tumor progression and therapeutic resistance [5].

Endothelial cells are essential components of the bone marrow niche, playing a significant role in the formation of blood vessels within the bone marrow, a process termed angiogenesis. Mesenchymal stem cells (MSCs) play a crucial role in angiogenesis associated with multiple myeloma, mainly by secreting pro-angiogenic factors like vascular endothelial growth factor (VEGF) and fibroblast growth factor 2 (FGF2). The endothelial cells in the BM respond to these signals by promoting the formation of new blood vessels, which are crucial for delivering oxygen and nutrients to the developing tumor. The role of MSCs in angiogenesis is notably dualistic. Mesenchymal stem cells (MSCs) can either promote angiogenesis, thereby facilitating tumor growth and metastasis, or contribute to the development of abnormal, permeable blood vessels that inadequately support tumor survival, depending on the specific conditions present. Examining the interactions among MSCs, endothelial cells, and angiogenesis is essential for elucidating the complexities of MM progression and therapeutic resistance [6].

The impact of MSCs on the immune microenvironment in multiple myeloma complicates their role in tumor progression. Mesenchymal stem cells (MSCs) interact with immune cells, such as T cells, dendritic cells, and natural killer cells, through the secretion of cytokines and extracellular vesicles. Such interactions may induce immune tolerance and suppress anti-tumor immune responses, a phenomenon that is particularly adverse in cancer contexts. In multiple myeloma, mesenchymal stem cells facilitate immune evasion through the secretion of cytokines, including IL-6 and CCL2, which enhance the survival and proliferation of multiple myeloma cells while suppressing immune cell activity. The function of MSCs in immune modulation is significantly influenced by the local microenvironment, disease stage, and external stimuli. A refined understanding of MSC-immune cell interactions in multiple myeloma is essential for developing targeted therapies that reverse immune suppression and enhance anti-tumor immunity [7].

MSCs can exert anti-tumor effects, particularly through the modulation of the immune microenvironment. MSCs influence immune cell functions by releasing cytokines and extracellular vesicles, which can induce immune tolerance and inhibit immune responses typically directed against MM cells, as shown in Fig. 1. The immunomodulatory properties of MSCs may aid in the prevention of autoimmunity or inflammation; however, they also impede the body’s inherent defence mechanisms against multiple myeloma (MM). Moreover, MSCs have demonstrated the ability to mediate resistance to chemotherapy and other treatments, thereby complicating therapeutic strategies [8, 9].

The therapeutic implications of mesenchymal stem cells in multiple myeloma are consequently intricate. Targeting MSCs may inhibit their pro-tumorigenic effects, potentially enhancing patient outcomes by decreasing tumour growth, metastasis, and drug resistance. Conversely, manipulating MSCs requires consideration of their essential functions in preserving bone homeostasis and immune tolerance, rendering MSC-targeted therapies a complex balancing act [10]. Comprehending the dual functions of MSCs in MM progression is crucial for the advancement of more effective and targeted therapies, potentially enhancing patient survival rates and quality of life.

Table 2 presents a summary of the conflicting findings related to the roles of MSCs in immune modulation and angiogenesis in MM, emphasizing the critical factors that lead to these inconsistencies. This indicates potential therapeutic implications that must be considered in the development of MSC-targeted therapies [11–13]. Also, we compare and contrast MSC properties between healthy and multiple myeloma bone marrow microenvironments in Table 1.

**Table 2:** Contradictory findings on the dual roles of mesenchymal stromal cells (MSCs) in immune modulation and angiogenesis in multiple myeloma, with key factors contributing to discrepancies and therapeutic implications

Aspect	Pro-Tumor Effects	Anti-Tumor Effects	Key Signaling Pathways	Relevant Biomarkers	Contradictory Findings	Contradictions Based on MSC Subpopulations	Contradictions Based on Disease Stages	Contradictions Based on External Stimuli	Potential Explanations for Discrepancies	Therapeutic Implications	Challenges	References
**Immune Modulation**	MSCs promote immune evasion by inducing Tregs and secreting TGF-β and IL-10. They suppress cytotoxic T-cell responses, creating an immunosuppressive microenvironment.	MSCs stimulate anti-tumor immunity by activating NK cells and promoting dendritic cell maturation.	TGF-β/SMAD, IL-10/JAK-STAT	TGF-β, IL-10, PD-L1	MSCs show both immunosuppressive and immune-activating roles depending on external stimuli.	Some MSC subpopulations (e.g., CD271 + MSCs) enhance immune suppression, while others (e.g., CD45- MSCs) activate immunity.	In early MM stages, MSCs promote immune suppression, while in advanced stages, they may support immune activation through NK cells.	MSCs may switch roles based on exposure to cytokines (e.g., IL-6, TNF-α) from MM cells.	MSC response varies by cytokine milieu and MM stage. Immune microenvironment interactions can drive conflicting roles.	Combining MSC-modulating agents with immune checkpoint inhibitors to reprogram immune suppression into immune activation.	Variability in MSC response to cytokines and immune cells; risks of autoimmunity when immune suppression is reversed.	[18]
**Angiogenesis**	MSCs secrete VEGF and IL-6, enhancing blood vessel formation to support MM growth.	Under certain conditions, MSCs secrete thrombospondin-1 (TSP-1), reducing angiogenesis.	VEGF/VEGFR, PI3K/AKT/mTOR	VEGF, TSP-1, Angiopoietin-1	Contradictory reports of MSCs promoting or inhibiting angiogenesis under varying hypoxic conditions.	CD73 + MSCs promote angiogenesis, while CD90 + MSCs may reduce angiogenesis through TSP-1.	MSCs in early MM stages may promote angiogenesis, while in later stages, MSCs may inhibit it through TSP-1 secretion.	MSCs exposed to hypoxic or inflammatory conditions can produce different angiogenic factors, contributing to conflicting findings.	Hypoxia-driven signaling or interactions with MM cells can tip the angiogenic balance.	Targeting angiogenesis via VEGF inhibitors or TSP-1 inducers combined with MSC-modulating drugs for synergy.	Tumor resistance via alternative angiogenic pathways, lack of specificity in angiogenesis inhibitors.	[19, 20]
**MSC Heterogeneity**	Some MSC subpopulations favor tumor growth by increasing immune suppression and angiogenesis.	Other subpopulations resist MM-induced changes and retain anti-tumor functions.	Notch, Wnt/β-catenin	CD73, CD90, CD105	Lack of standardized methods to isolate and characterize MSC subsets leads to conflicting findings.	MSCs derived from different anatomical sites (e.g., bone marrow vs. adipose tissue) exhibit distinct pro-tumor or anti-tumor behaviors.	MSCs in earlier stages of MM may maintain a more anti-tumor phenotype, while later stages of MM lead to MSC reprogramming that drives tumor progression.	The presence of inflammatory cytokines and external factors (e.g., chemotherapy) can alter MSC behavior, leading to conflicting outcomes.	MSC plasticity under therapy-induced changes could drive unexpected responses, highlighting the need for patient-specific approaches.	Therapeutics targeting specific MSC subpopulations based on their tumor-promoting or anti-tumor properties.	Variability in MSC characteristics across patients; difficulty isolating and targeting specific subpopulations.	[21]
**Impact of MM Niche**	MM cells reprogram MSCs via cytokines (e.g., IL-6, TNF-α), making them secrete tumor-promoting factors and adopt a pro-tumor phenotype.	Certain MSCs resist reprogramming, maintaining anti-tumor cytokine secretion and immune stimulation.	TNF-α/NF-κB, IL-6/JAK-STAT	IL-6, TNF-α, CXCL12	Some studies show MSC resistance to MM reprogramming, while others report complete MSC functional changes under MM influence.	Bone marrow-derived MSCs (BM-MSCs) are more susceptible to MM reprogramming compared to MSCs from other tissues.	In early MM stages, MSCs resist reprogramming and maintain anti-tumor function, while in advanced stages, they become more tumor-supportive.	MM-induced cytokine storms (e.g., IL-6) dramatically shift MSC functions towards promoting tumor growth, resulting in discrepancies.	Differences in MM subtypes or niche microenvironments likely influence MSC reprogramming capabilities.	Blocking MM cell-MSC interactions (e.g., CXCR4 inhibitors) to prevent MSC reprogramming and tumor-supportive functions.	Heterogeneity in MM-MSC interactions; redundancy in signaling pathways may reduce therapeutic efficacy.	[22]
**Therapeutic Targeting**	MSCs can drive resistance to therapies such as proteasome inhibitors and immunomodulatory drugs by secreting survival factors.	Modifying MSCs or targeting their secretions (e.g., with exosome-based therapies) can enhance anti-tumor responses.	NF-κB, IL-6/JAK-STAT, PD-1/PD-L1	IL-6, PD-1, PD-L1	Dual effects of MSC-targeted therapies on tumor progression versus immune regulation create challenges for clinical translation.	MSCs in early MM may be more sensitive to therapy, while in later stages, they may show resistance mechanisms.	In early stages, MSCs may promote the anti-tumor efficacy of chemotherapies, while in advanced stages, they may reduce the efficacy through immunosuppressive effects.	MSCs exposed to chemotherapy or targeted therapies could switch from pro-tumor to anti-tumor functions, depending on the drugs used.	MSC plasticity makes them unpredictable in their therapeutic response.	Combining MSC-targeting drugs with immune therapies or angiogenesis inhibitors to exploit their multi-faceted roles in the tumor microenvironment.	Long-term safety concerns with MSC-modulating therapies; risks of targeting normal bone marrow MSCs.	[23]
**MSC-Derived Exosomes**	MSC exosomes may carry pro-tumor molecules like VEGF, promoting MM growth.	Engineered MSC exosomes loaded with siRNA or anti-MM molecules can deliver targeted therapies.	PI3K/AKT, MAPK	VEGF, miR-155	Exosome heterogeneity complicates therapeutic applications; pro- and anti-tumor effects depend on cargo and source.	Exosomes from different MSC subpopulations exhibit different bioactivities.	In early MM stages, MSC-derived exosomes may have anti-tumor effects, while in advanced stages, they may be pro-tumor.	Exosome behavior varies based on the tissue origin of MSCs and external factors such as inflammation or hypoxia.	Exosome content is influenced by MSC state and external stimuli; engineering can reduce variability.	MSC-derived exosome therapy offers a novel, minimally invasive treatment option but requires fine-tuning for consistent efficacy.	Manufacturing scalability, stability of engineered exosomes, and delivery specificity remain major hurdles.	[24]

This review will discuss the documented contributions of MSCs to immune checkpoint inhibitors (ICIs), pro-inflammatory functions, tumor epigenetics, reprogramming, chemotherapy resistance, and senescence, among other aspects of muscular dystrophy pathology. We will explore how these interactions influence the development and progression of MM.

Figure 1 depicts the interaction between multiple myeloma cells and mesenchymal stromal cells in the bone marrow niche. This figure illustrates the processes by which MSCs influence tumour progression and immune modulation, offering a visual representation of their intricate role in the disease. This review examines the conflicting evidence concerning the roles of MSCs in immune modulation, angiogenesis, and tumour progression in multiple myeloma, highlighting the necessity for a more refined approach to MSC-targeted therapies. This discussion will focus on the key factors contributing to discrepancies, offering insights into how emerging technologies can elucidate the complexities of MSC-MM interactions and propose innovative experimental approaches for future research [14–17].

The variability in mesenchymal stem cell (MSC) function observed in various studies is largely attributed to the intrinsic heterogeneity of MSC populations. Despite widely recognized defining criteria plastic adherence, expression of CD73, CD90, and CD105, and trilineage differentiation potential MSCs display significant variability in their molecular and functional characteristics. Recent studies using single-cell RNA sequencing have shown that MSCs comprise various subpopulations with unique transcriptomic profiles, each exhibiting different immunomodulatory, pro-angiogenic, and regenerative abilities [25–27]. Bone marrow-derived MSCs (BM-MSCs) and adipose-derived MSCs (AD-MSCs) demonstrate distinct cytokine secretion profiles, with BM-MSCs preferentially producing IL-10 and AD-MSCs showing a more significant TNF-α response. Aging and disease conditions significantly impact MSC function. In multiple myeloma (MM), MSCs from patients show altered osteogenic differentiation potential, increased secretion of pro-inflammatory cytokines such as IL-6 and CCL2, and enhanced support for tumor growth. The modifications induced by diseases lead to inconsistent results in MSC research, underscoring the necessity for more accurate characterization of MSC subsets prior to their therapeutic use [28].

The microenvironment significantly influences MSC function, with minor variations in external stimuli resulting in distinct outcomes, in addition to intrinsic heterogeneity. Inflammatory cytokines, including TNF-α, IFN-γ, and IL-1β, are recognized as significant modulators of MSC behavior; nonetheless, their effects are notably context-dependent. Preconditioning MSCs with IFN-γ improves their capacity to inhibit T-cell proliferation in inflammatory conditions such as graft-versus-host disease (GvHD). However, in the presence of IL-1β, MSCs can shift to a pro-inflammatory phenotype, which may worsen tissue damage [29]. Hypoxia, a prevalent characteristic of the bone marrow niche in multiple myeloma patients, can markedly affect mesenchymal stem cell function. Moderate hypoxia (1–5% O₂) increases MSC secretion of VEGF and FGF2, thereby facilitating angiogenesis and tissue repair. In contrast, severe hypoxia (< 1% O₂) triggers oxidative stress, resulting in cellular senescence and diminished regenerative capacity. In multiple myeloma, mesenchymal stem cells are subjected to chronic hypoxia, which has been demonstrated to alter their function in favor of tumor progression by elevating the secretion of pro-angiogenic factors and upregulating adhesion molecules that promote the survival of multiple myeloma cells. This highlights the necessity of thorough examination of microenvironmental factors when analyzing inconsistent MSC data across research studies [30].

Disease-specific factors complicate the functionality and therapeutic potential of MSCs. In autoimmune diseases like rheumatoid arthritis (RA), mesenchymal stem cells (MSCs) demonstrate both immunosuppressive and pro-inflammatory effects, which vary according to the disease stage and cytokine environment. In early-stage rheumatoid arthritis (RA), mesenchymal stem cells (MSCs) primarily inhibit T-cell activation and decrease joint inflammation. Conversely, in chronic RA, MSCs may lose their immunosuppressive capabilities due to extended exposure to inflammatory mediators, leading to a senescent and dysfunctional phenotype [31]. In multiple myeloma, mesenchymal stem cells demonstrate significant changes relative to their healthy equivalents. MSCs associated with multiple myeloma exhibit diminished osteogenic differentiation, elevated secretion of IL-6 and CCL2, and increased facilitation of myeloma cell proliferation and drug resistance. Ullah et al. [32]. conducted a study indicating that MSCs from MM patients increase the expression of CXCL12, a chemokine essential for the homing of myeloma cells to the bone marrow, which contributes to the acceleration of disease progression. The findings indicate that MSC function is dynamic and varies according to disease context, potentially accounting for the inconsistent outcomes of MSC-based therapies across various conditions [33].

Future research must focus on a mechanistic approach to MSC characterization, incorporating multi-omics technologies like single-cell RNA sequencing, proteomics, and metabolomics to accurately define functional MSC subtypes. Furthermore, standardizing pre-conditioning protocols, including the optimization of oxygen tension, cytokine priming, and epigenetic modifications, could reduce variability in MSC function and enhance reproducibility. In multiple myeloma, therapeutic strategies should focus on reversing the tumor-supportive phenotype of mesenchymal stem cells, potentially via targeted inhibition of IL-6 or CXCL12 signaling. Additionally, utilizing MSCs from healthy donors instead of patient-derived MSCs may improve therapeutic efficacy, given that diseased MSCs frequently demonstrate impaired function. A comprehensive understanding of the molecular mechanisms that regulate MSC behavior across various disease contexts is essential for the advancement of more predictable and effective MSC-based therapies [34].

## Methods

A comprehensive search of the PubMed, Scopus, Web of Science and Google scholar was performed from database inception to March 2024. The key word used for search included Multiple myeloma, Bone marrow mesenchymal stromal cells and Myeloma progression. The literature inclusion criteria were as follows: (I) reports involving the roles of MSCs in various aspects of MM pathology, including their pro-inflammatory functions, involvement in tumor epigenetics, effects on immune checkpoint inhibitors (ICIs), impact on reprogramming, chemotherapy resistance, and senescence; (II) the article was an original study; (III) the language of the literature was English. The exclusion criteria were as follows: article types such as conference abstracts, case reports, non-English, case series and letter. The searches were conducted by 2 researchers individually. After removal of duplicate literature, the articles were screened according to the relevance of the content. Different opinions or results were resolved through negotiation between the 2 researchers.

### Metabolism in the bone microenvironment cells

The specialized cells known as osteoblasts (OBs) are involved in the process of bone production, while osteoclasts (OCs) are responsible for the resorption process. Bone remodeling mechanisms become uncoupled in MM, leading to the development of osteolytic bone lesions. An increase in osteoclastic formation and a decrease in osteoblastic formation describe altered bone remodeling [42, 43]. In fact, both the modeling and remodeling processes necessitate the manufacture of collagen and other matrix proteins by osteoblasts (OBs), thereby consuming a substantial quantity of energy in the form of adenosine triphosphate (ATP) [44, 45]. Given this, it is not unexpected that changes in the metabolite availability in the MM microenvironment produce modifications in the energy metabolism of bone cells, which in turn affect their development and function [46, 47]. Furthermore, new research has shown that the WNT pathway directly alters cellular metabolism in OB lineage cells by promoting Gln catabolism, fatty acid oxidation, and aerobic glycolysis [48]. In other words, cancer cells primarily use glucose, fatty acids, and amino acids to meet their biosynthetic needs [49–52]. Glucose is one of the most important fuel substrates for OBs. It enters cells through glucose transporters (GLUTs) through facilitated diffusion, an energy-free mechanism [53]. The main glucose transporter in OBs seems to be glucose transporter 1 (GLUT1), which is encoded by SLC2A1. However, GLUT3 and GLUT4 are also expressed [54]. As stated, before that OBs and their progenitors experience extensive energy reprogramming throughout the differentiation process. Research by Guntur et al. demonstrated that oxidative phosphorylation is the preferable method following the start of differentiation and matrix synthesis, but glycolysis is the primary means by which OB progenitors in mice produce ATP. Once mineralization is complete, mature OBs lean towards glycolysis. In line with these findings, metabolic tracing investigations demonstrated that the majority of glucose in mature OBs is converted to lactate and that glycolysis is the primary mechanism for ATP generation [55–57]. Tumor cells exhibit metabolic reprogramming, referred to as the Warburg Effect, which is believed to facilitate the production of amino acids, nucleotides, and lipids required for cell division. Moreover, the augmentation of aerobic glycolysis could aid in the mitigation of reactive oxygen species and additionally facilitate the production of a greater quantity of amino acids to support protein synthesis in osteoblasts [58]. Finally, glucose transport and metabolism could potentially be affected by the anti-MM medicines that are now being utilized. According to reports, when MM cells are treated with vincristine or the proteasome inhibitor bortezomib, the expression of GLUT1 and HK2 (Hexokinase 2) is reduced, leading to apoptosis [59]. OBs can utilize amino acids for the process of protein synthesis, or alternatively, they can undergo metabolism to produce ATP, which serves as a source of energy [60]. Glutamine (Gln), a nonessential amino acid (NEAA), is primarily produced by the enzyme Gln synthetase (GS) using glutamate (Glu) and ammonia (NH3) as substrates. It has been recognized as a significant regulator of osteoblasts due to the increased demand for amino acids resulting from the augmented synthesis of the bone matrix during bone formation [61]. Gln is transported into the cells through plasma-membrane Gln transporters, specifically SLC1A5 (ASCT2), SLC7A7, and SLC38A2 (SNAT2) [46, 62]. During the differentiation process of BM stromal cells (BMSCs) into osteoblasts, a significant amount of Gln is consumed. However, this consumption of Gln does not occur when BMSCs convert into adipocytes. According to reports, Gln enhances the activity of GLS (glutaminase) and GDH (Glutamate dehydrogenase) through the mTOR/S6 and MAPK signaling pathways, therefore stimulating cell proliferation [63]. Due to the rapid proliferation of OB progenitors into mature OBs capable of synthesizing bone matrix, the differentiation process is marked by a heightened use of Gln. It has been observed that when osteoblasts are induced to undergo mineralization, glucose alone is inadequate to fulfill their energy needs. The degree of mineralization only increases when cells are provided with glucose and glutamine supplementation [64, 65]. Many studies have shown that OBs and progenitors use different amino acids such as proline, methionine, arginine and tryptophan in their metabolism to support anabolic functions during bone formation and mineralization [49]. Recent investigations have shown that MM cells restrict the formation of osteoblasts by impeding the synthesis of asparagine in mesenchymal cells through the depletion of glutamine. This provides a metabolic explanation for the suppression of osteoblasts in MM. Ultimately, the metabolic characteristics of malignant plasma cells result in the creation of a bone marrow microenvironment that is distinguished by low amounts of Gln and high levels of Glu. This leads to a decrease in the development of osteoblasts and an increase in the differentiation of osteoclasts. Hence, directing attention towards the Gln glutamate axis may serve as an innovative therapeutic strategy for bone disease in patients with multiple myeloma [66].

### The interplay between mesenchymal stromal cells and immune cells in TME

MSCs by secretion of bioactive molecules or directly interacting with endothelial cells, cancer cells, and immune cells modulate the immune cell response with anti-inflammatory effects and pro-inflammatory or anti-tumor effects. The roles of MSC in TME are complex, so they can both promote inflammation or form new tumors and or inhibit tumor progression by modulating the innate and adaptive immune response and anti-inflammatory effects [67–71]. Owing to their varied characteristics, MSCs are probably found in TME, in multiple subpopulations, including naive MSCs and tumor-derived MSCs (T-MSCs), which have distinct roles within the TME. While T-MSCs solely encourage tumor progression, naive MSCs simultaneously prevent and promote tumor progression [72, 73]. New insights into the inflammatory milieu, tumors, and MSCs’ mechanisms of interaction have been revealed. These insights could help us better understand the initiation or progression of cancer. Tissue damage and lesions in cancer sites make a pro-inflammatory environment that recruits various cells such as MSCs [74–76]. The migration of MSC to the TME is regulated by numerous factors, including adhesion molecules (VCAM), chemokines (CCL2, CCL5, CCL22, CXCL8, and SDF-1/CXCL12), cytokines (IL-1, IL-6, TGF-β, and TNF-α), insulin GF (IGF-1), PDGF, hypoxia-inducible factor (HIF-1), reactive oxygen species (ROS), and many more [77]. MSCs use a variety of methods to promote and inhibit inflammation. In innate immunity, BM-MSCs express C3aR and C5aR (Complement system receptors), as anaphylatoxin receptors, for recruitment of MSCs to the sites of damage or lesions [72, 78, 79]. MSCs by secretion of CXCL3, HGF, and IL-6 have a role in myeloid suppressor cell (MDSC) differentiation and in this way suppress the immune system in TME. One of the recruited cells to the inflammation sites are macrophage [79]. MSCs by secretion of IDO, IL-6, and PGE2 participate in the differentiation of macrophages to immunosuppressive M2 (tissue repair-associated) phenotype [80–82]. The adaptive immune system is affected by MSCs in several ways. The maturation and activation of both immature and mature dendritic cells (DC) are affected by MSCs [83]. MSCs cause immature DCs to exhibit a diminished capacity to present antigen, encourage T cell proliferation, and differentiate naive T-cells, which results in inefficient T cell activation [84]. Through IL-10, PD-1, and PGE2, MSCs prevent T cell development into T helper (Th)1 and Th17 (pro-inflammatory) cells [85, 86]. Hence, assessing the immunosuppressive qualities of MSCs is frequently utilized as a functional assay to determine the potency of MSCs. In fact, MSCs are being used as a cellular therapy for inflammatory and degenerative diseases, where B cells have pathogenic roles, either in clinical trials or approved for use in the clinic. In this regard, Porter et al. conducted a study and finally showed that MSCs block the production of matrix antibody responses which include primary (IgM), secondary (IgG isotypes), and mucosal (IgA) antibody responses. In addition, it is shown in this study that that MSCs effectively block B cell matrix responses when PBMCs are used but not purified B cells [87]. However, previous studies have shown different results in this field. In fact, it is reported in these studies that MSCs promote the survival of plasma cells, B cells and support antibody secretions [88–90] (Table 3).

**Table 3:** This table illustrates the the interplay between mesenchymal stromal cells and immune cells in TME

Aspect	Mesenchymal Stromal Cells (MSCs)	Immune Cells (TME Interaction)	Effects on Tumor Progression	Mechanisms of Action	Secreted Molecules / Receptors	Impact on Innate Immunity	Impact on Adaptive Immunity	Other Factors	References
**Role in TME**	MSCs can both promote and inhibit tumor progression	Interact with immune cells (e.g., NK cells, macrophages, T-cells, DCs) and endothelial cells	MSCs promote inflammation, suppress immune response, or inhibit tumor growth	MSCs secrete bioactive molecules and directly interact with immune cells	PGE2, TGF-β, IDO, IL-6, CXCL3, IL-10, PD-1	MSCs modulate NK cell cytotoxicity and cytokine production	MSCs affect dendritic cell (DC) maturation and antigen presentation	MSCs respond to hypoxia, adhesion molecules (VCAM), and cytokines (IL-6, TNF-α)	[91]
**MSC Subpopulations**	Naive MSCs: Promote and inhibit tumor progression	Interact with macrophages, NK cells, MDSCs, T cells	Tumor-derived MSCs (T-MSCs) promote tumor growth	Modulate innate and adaptive immune responses through secretion of cytokines and immune ligands	IDO, IL-10, HLA-G, CCL2, CXCL12	NK cell activity inhibited by PGE2, IDO, TGF-β, HLA-G secretion	MSCs prevent T-cell proliferation and promote Treg differentiation	MSC migration regulated by HIF-1, chemokines (CCL2, CXCL8)	[92]
**Effect on NK Cells**	Inhibit NK cell proliferation and cytotoxicity	NK cells destroy MSCs via activating ligands (MICA, CD112)	MSCs block tumor progression by inhibiting NK cells	Suppression of NK cell cytotoxicity via soluble factors	PGE2, IDO, HLA-G, TGF-β	MSCs express UL16 binding proteins, activating NK receptors for immune response regulation	MSCs suppress NK cytotoxicity via PGE2 and HLA-G secretion	Tregs are promoted by MSC secretion of IL-6 and IL-10	[93]
**Effect on Macrophages**	Promote immunosuppressive M2 macrophage differentiation	MSCs influence macrophage function in inflammatory sites	MSCs suppress immune responses, aiding tumor immune evasion	Secretion of cytokines like IL-6 and IDO to promote M2 phenotype	IDO, PGE2, IL-6, HGF	MSCs induce M2 macrophages for tissue repair and immune suppression	T-cell proliferation is inhibited via ICAM-1-LFA-1 interaction	MSCs promote Th2 responses and suppress Th1/Th17 cells through IL-4	[94]
**Effect on Dendritic Cells (DCs)**	MSCs inhibit DC maturation and antigen presentation	Dendritic cells have reduced capacity to activate T cells under MSC influence	MSCs cause inefficient T-cell activation in TME	Secretion of TGF-β, PGE2, and IL-10, blocking DC function	TGF-β, PGE2, IL-10	DCs under MSC influence have decreased capacity to present antigens	MSCs inhibit T-cell proliferation through cell-cell interactions	MSCs promote immune tolerance by inducing Tregs	[95]
**Effect on T Cells**	Inhibit T-cell proliferation and activation	Tregs promoted, Th1/Th17 cells suppressed in TME	MSCs suppress tumor progression by modulating T-cell response	Secretion of IL-10, IL-4, and TGF-β, promoting Treg differentiation	IL-10, PD-1, TGF-β	MSCs inhibit T-cell proliferation via IFN-γ and ICAM-1–LFA-1 signaling	MSCs promote Treg differentiation through IL-6, IL-10	MSCs suppress inflammation by promoting Treg cells	[96]
**Effect on B Cells**	Suppress antibody responses in inflammatory diseases	Promote B-cell survival and plasma cell antibody production	MSCs suppress the immune system in TME, aiding tumor evasion	Block matrix antibody responses (IgM, IgG, IgA)	IL-6, IL-10, TGF-β, IDO	MSCs inhibit matrix antibody response when PBMCs are used but not purified B cells	MSCs block B-cell antibody responses in PBMC environments	MSCs support B-cell antibody secretion and plasma cell survival	[97, 98]

### Pro-inflammatory role of mesenchymal stromal cells on the progression of MM cells

Increases in proliferation, homing patterns, and survival are the results of the interaction between myeloma cells and BM stromal cells. Furthermore, it has the ability to initiate both autocrine and paracrine mediator secretion, including cytokines induced by INF-gamma, HGF, IL-2, IL-16, and EGF. In addition to the presence of inflammatory cells within the TME, key non-tumor cells found therein include inflammatory cells like macrophages, lymphocytes, myeloid-derived suppressor cells, vascular endothelial cells, and tumor-associated stromal cells like MSCs and tumor-associated fibroblast (TAF). These non-tumor cells play a critical role in the progression of tumors, and defects in MSC lead to hematopoietic abnormalities [99]. On their surface, MM cells express VLA4, LFA1, MUC1, or CD40 [100]. In BM, MM cells attach to type 1 collagen, fibronectin, and hyaluronan via syndecan, CD138, VLA4, and CD44, respectively [101]. The activation of NF-KB, which is the outcome of the VLA4-VCAM1 molecule in MM cells and MSCs, stimulates the transcription and release of the primary MM cell growth molecule, IL-6 [102]. Moreover, VEGF and BFGF are produced and secreted by MM cells in a more favorable manner by IL6, and they both bind to receptors on MSC to encourage the production of IL-6 [101, 103]. Moreover, by encouraging MM-MSCs’ proliferation, chemoresistance, and immune escape, NF-κB (Nuclear factor Kappa B) plays a critical role in controlling the impact that MM-MSCs have on MM cells [104]. According to recent studies, MSCs have been shown to influence different immune cells, including DCs, NK cells, lymphocytes, and monocytes. This effect is caused by a variety of substances, including enzymes, adenosine, chemokines, and cytokines [105, 106]. MSC promotes the transition of macrophages from the M1 to the M2 phenotype as part of innate immunity by enhancing anti-inflammatory cytokines like IL-10 and preventing the release of pro-inflammatory cytokines like TNF-alpha and INF-gamma. Although MSCs have little effect on the formation of active NK cells, they can inhibit the growth of dormant NK cells and alter their properties and functions by direct interaction or the production of chemicals including IL-2, TGF-β, and PGE2. Furthermore, MSC impairs the development of DCs from monocytes or hematopoietic precursors and stops them from secreting pro-inflammatory cytokines like TNF-α, which suppresses the polarization of Th1 cells. MSC also directsDC to develop a tolerant phenotype and promote the development of Treg cells and Th2 cells [107]. Previous research found that TNF-α-activated MSCs inhibit inflammation by provoking the synthesis of IL10 in macrophage cells. However, MSCs themselves do not produce IL-10, as MSCs from IL-10-/- mice nonetheless effectively improved the survival of mice with sepsis. On the contrary, MSCs produce TGF-β, which acts as an immunosuppressive molecule. This TGF-β also has a function in boosting the production of IL-10 by T cells. It does so by directly activating the IL-10 promoter through Co-Smad4. When exposed to a certain amount of TNF-α, MSCs will be activated and transformed into an immune-suppressive type called MSC type 2. This transformation occurs because the MSCs release different anti-inflammatory molecules, such as TGF-β and IL-10. Nevertheless, it remains uncertain whether MSCs possess the capacity to generate IL-10 when exposed to TNF-α activation. The ability of MSC to produce TGF beta and IL-10 at 5 and 10 ng/ml following a 24-hour incubation period was assessed in a study. The findings indicated a noteworthy rise in TGF beta and IL-10 levels at a 5 ng/ml dose of TNF-alpha relative to a 10 ng/ml dose of TNF-alpha. Thus, TNF-alpha at a dosage of 5 ng/mL is adequate for MSC to suppress inflammation [108].

Recently, treating MM with MSC has been a new trend in stem cell research, however, the effect of MSC infusion on suppressing or progressing MM is controversial. According to in vitro research, MM patent MSC contains aberrant genetic, phenotypic, and functional traits that might cause defects in bone production while shielding MM cells from apoptosis [106, 109]. Moreover, newer studies have depicted that injection of MSC subcutaneously develops tumor growth and neovascularization in syngeneic mouse models by promoting tumor vasculature and secreting proangiogenic factors [110]. Accordingly, MSCs have an important role in tumor progression, whether they play a role as a tumor suppressor or a tumor promoter [111] (Table 4).

**Table 4:** Pro-inflammatory role of mesenchymal stromal cells on the progression of MM cells in depth

Aspect	Mesenchymal Stromal Cells (MSCs)	Myeloma (MM) Cells	Tumor Microenvironment (TME)	Inflammatory Mechanisms	Key Cytokines / Molecules	Effect on Immune Cells	Impact on Tumor Progression	Effect of MSC Infusion	References
**Interaction with MM Cells**	MSCs promote proliferation, homing, and survival of MM cells	MM cells express surface molecules (e.g., VLA4, LFA1, MUC1)	TME contains inflammatory cells (macrophages, lymphocytes, endothelial cells, MDSCs)	MSCs activate NF-κB via VLA4-VCAM1 interaction, inducing IL-6 secretion	IL-6, TNF-α, IL-1β, INF-γ, HGF, IL-2, IL-16, EGF	MSCs inhibit NK cell proliferation and DC differentiation; promote M2 macrophages	MSCs promote chemoresistance, proliferation, and immune escape of MM cells by activating NF-κB	MSCs may promote tumor growth and neovascularization in mouse models	[112]
**Role in Cytokine Secretion**	MSCs enhance both autocrine and paracrine cytokine secretion	MM cells secrete VEGF and bFGF, which bind to MSC receptors	MSCs respond to inflammatory signals from MM cells and other TME cells	MSCs induce IL-6 production, which supports MM cell survival and growth	IL-6, VEGF, bFGF, TNF-α, INF-γ, PGE2, TGF-β	MSCs suppress NK cells, T cells, and DCs; promote immunosuppressive M2 macrophages	MSCs promote angiogenesis, supporting MM growth	In vitro studies show that MSCs protect MM cells from apoptosis	[113, 114]
**Effect on Inflammatory Pathways**	MSCs activate NF-κB-dependent inflammatory pathways	MM cells activate MSCs to produce more inflammatory cytokines	NF-κB activation by TLR4 ligands and TNF-α/IL-1β leads to inflammation	NF-κB activation promotes MM-MSC proliferation, survival, and chemoresistance	NF-κB, IL-6, TNF-α, IL-1β	MSCs prevent the development of pro-inflammatory Th1 cells and promote Th2 cells	MSCs contribute to tumor progression by immune modulation	MSC-derived factors can promote immune escape and angiogenesis in MM	[29, 115]
**Effect on Macrophages**	MSCs promote M1-to-M2 macrophage transition (immunosuppressive)	MM cells induce MSC secretion of IL-10, TGF-β	MSCs influence TME by promoting anti-inflammatory M2 macrophages	MSCs increase IL-10, TGF-β secretion to suppress inflammation	IL-10, TGF-β, TNF-α, PGE2	MSCs shift macrophage phenotype to M2, which supports tumor growth and tissue repair	M2 macrophages promote tumor growth and immune evasion	MSCs inhibit pro-inflammatory cytokine release from macrophages, aiding MM survival	[116, 117]
**Effect on NK Cells**	MSCs inhibit NK cell proliferation and cytotoxicity	MM cells evade NK cell immune response via MSC modulation	MSCs impair NK cell activity, promoting tumor immune evasion	MSCs secrete PGE2, IL-2, and TGF-β to suppress NK cell function	PGE2, IL-2, TGF-β, HGF	MSCs prevent NK cell maturation and suppress their cytotoxicity	Suppressed NK cell activity allows MM cells to evade immune surveillance	MSCs inhibit NK cells through direct interaction or secretion of cytokines	[118, 119]
**Effect on Dendritic Cells (DCs)**	MSCs impair DC development from monocytes and precursors	MM cells influence MSCs to modulate immune responses	MSCs prevent DCs from secreting pro-inflammatory cytokines like TNF-α	MSCs promote a tolerant DC phenotype, reducing pro-inflammatory cytokines	TNF-α, IL-10, TGF-β	MSCs inhibit DC maturation, resulting in suppressed T-cell activation	Tolerant DCs allow MM cells to proliferate by evading immune detection	MSC-induced DC tolerance supports immune evasion of MM cells	[120, 121]
**Effect on T Cells**	MSCs inhibit T cell proliferation and promote Treg cells	MM cells benefit from MSC-mediated immune suppression	MSCs promote anti-inflammatory Treg cells, suppressing Th1/Th17 cells	MSCs suppress T cell activation via IL-10, IL-4, and TGF-β secretion	IL-10, IL-4, TGF-β, PGE2	MSCs promote Treg differentiation, suppress Th1/Th17 cells	Suppressed T cell responses allow MM cells to evade immune control	MSC-mediated T cell suppression promotes MM growth by reducing immune responses	[122, 123]
**Controversy of MSC Infusion in MM**	MSCs have a dual role: suppressing and promoting tumor progression	MM-derived MSCs show abnormal traits that promote MM cell survival	MSCs play a controversial role in both tumor suppression and progression	MSCs protect MM cells from apoptosis and promote bone defects	VEGF, bFGF, IL-6, TGF-β, IL-10	MSCs promote tumor vasculature and angiogenesis, enhancing MM progression	MSCs may promote tumor growth and neovascularization in vivo	Some studies show MSCs shield MM cells from chemotherapy-induced apoptosis	[124–126]

### The effects of epigenetic mechanisms on the interaction of mesenchymal stromal cells and MM cells

It has been demonstrated that transferase and demethylase regulate distinct transcription factors, chromatin structure and genomic stability, X chromosome inactivation, genomic imprinting, and carcinogenesis about the differentiation and maturation of BM-MSC. We refer to this as epigenetic [127–129]. Epigenetics commence with the interconnection of DNA and histone proteins, which are two complex macromolecules forming the structure of chromatin. The fundamental structural unit of chromatin, known as the nucleosome, consists of repetitive 146-base-pair lengths of DNA that are coiled around a group of eight histone proteins. The histone protein family comprises H2A, H2B, H3, H4, and other variations, each with distinct activities. Histone proteins and DNA modifications are essential for controlling the accessibility and functionality of these molecules. Any alterations to these adjustments are regarded as noteworthy [130]. Epigenetic regulation refers to changing phenotypes by varying the expression of DNA sequence without altering its sequence or its coding. has the ability to be inherited, Epigenetic changes are the consequence of cell responses to extrinsic inheritance in order to enhance extrinsic environmental impetus and cellular intrinsic inheritance to control cell and niche hemostasis [131]. These types of alteration include methylation of DNA, changes of histones (acetylation, methylation, phosphorylation, ubiquitination, and sumoylation), and non-coding RNAs, such as RNA interacting with piwi (piRNA), microRNA (miRNA), long non-coding RNA (lncRNA), and small interfering RNA (siRNA) [132, 133].

MM can be the result of DNA hypomethylation and hypermethylation of specific gene promoters, which are important regulators in B cell differentiation [134]. In the DNA methylation mechanism, a methyl group to carbon5 of cytosine is catalyzed by methyltransferase. The sequence 5-CG-3 is known as GPC Island [135]. Prior research has indicated that MSCs derived from MM patients exhibit normal cytogenetic characteristics. However, these cells display changes in their transcriptional and proteomic profiles, even in the absence of observable chromosomal abnormalities. Because myeloma cells talk to each other, epigenetic processes may be in charge of how MSCs promote tumor growth and how they stop osteoblasts from dividing in MM. Adamik and colleagues discovered that there is an aberrant activation of chromatin remodelers in MSCs from myeloma patients. This abnormal activation ends with the suppression of Runx2, a key regulator of osteoblast development. However, there is a research gap in the pathways associated with DNA methylation that could potentially contribute to the progression of MM and subsequent bone abnormalities [136]. In research by Antonio Garcia-Gomez, MM-MSCs exhibit control over their tumor-promoting activity and prolong osteoblast suppression. It was found that BM -isolated MSCs from different stages of MM had widespread DNA methylation changes [137]. These changes were especially seen in Homeobox genes that play a role in osteogenic differentiation and are linked to these changes in DNA methylation can also be seen in the lab when MSCs from healthy people are exposed to MM cells [138]. Furthermore, it has been demonstrated that methylation contributes to the advancement of the MM stage. Demethylation predominantly takes place in monoclonal gammopathy of unknown significance (MGUS) and CpG islands during non-malignant stages. A notable decrease in methylation is the pivotal aspect of the shift from MGUS to MM. Moreover, methylation changes take place during the malignant stages [139]. DMNT3 is underexpressed as a result of its hypermethylation. Consequently, it is noteworthy that MM-staged cells appear to have DNA hypermethylation in B-cell-specific enhancers as a critical feature. Because the hypermethylated area is located at the binding sites of B-cell-exclusive transcription factors, it reduces the production of these factors, which in turn results in a less specialized cell profile in MM cells. Hypermethylation has been seen in stem cells and happens in enhancers specific to B cells. It is progressively eliminated in B cells that are not malignant, and it is then restored in MM cells [140]. In this regard, in a study by Garcia Gomez and colleagues on pharmacological targeting of DNMTs and G9a with the dual inhibitor CM-272, they showed that CM-272 treatment prevented tumor-associated bone loss and reduced tumor burden in the murine myeloma model. They concluded that epigenetic abnormalities in patients with MM lead to bone formation disorders and targeting it by CM-272 can reverse the process of Myeloma associated bone disease [138]. DNA hypermethylation at the CpG islands connected to the promoters of multiple tumor-suppressive genes, including CDKN2B, CDKN2A, CDH1, DAPK1, SOCS1, and SHP1, disturbed their activity. When it comes to bone problems linked to myeloma, BM-MSCs experience defective epigenetic remodeling. Because of the aberrant gene expression patterns, this promotes the formation of tumors and prolongs the suppression of osteoblasts (MBD). There is evidence of significant amounts of DNA methylation alteration, especially in homeobox genes, which influence aberrant expression and lead to osteogenic differentiation [141]. By altering these genes’ promoters, the demethylation process changes how osteogenesis regulates gene expression. According to this perspective, the primary driver of osteoblast differentiation has been identified as the HOX family [142]. Therefore, it has been noted that DNA methylation is important for identifying the lineage of MSCs, as well as for tumor growth and immune system suppression in different kinds of cancer [143–146]. The lysine acetylation forward and backward processes are catalyzed, respectively, by histone acetyltransferase [110] and histone deacetylases (HDACs) [147]. HDACs modify gene transcription, chromatin structure, protein-DNA interactions, and histone tail length as epigenetic regulators. HDAC and HAT also target other proteins, such as those involved in the DNA damage response, hormone receptors, transcription factors, chaperone proteins, and signaling mediators [148]. This results in a multitude of hematological malignancies because of histone’s crucial involvement in genomic regulation. For example, lymphomas and leukemias exhibit the following changes:

(1) There are alterations in the general acetylation pattern in cancer cells. (2) Lymphoma cells have higher HDAC levels. (3) Aberrant attraction of HDAC to a target gene results in transcription suppression and differentiation inhibition, which in turn contributes to the development of acute promyelocytic leukemia [149, 150].

HDAC inhibitor vorinostat was utilized to treat HMCs in research by Song XU et al. While apoptosis was seen after exposure to greater doses of vorinostat (10 and 50 µmol/L), it was shown that the low concentration of vorinostat (1 µmol/L) did not significantly increase apoptosis in hMSCs. hence the anti-myeloma medication, but no decrease in bone growth was seen [151]. Ahmadvand et al. demonstrated that VEGF gene expression might be lowered in MSCs through HDAC-mediated downregulation of chromatin remodeling. Thus, during tissue regeneration, chromatin modification might be a possible mechanism that influences MSC paracrine and subsequent angiogenesis [152]. In this regard, Adamik et al., showed that the repressive chromatin architecture at Runx2 was reversed and osteoblast differentiation was salvaged when EZH2 or HDAC1 activity was inhibited in pre-osteoblasts following multiple myeloma exposure in vitro or in osteoblast precursors from patients with multiple myeloma [136]. MSC-targeted therapies represent a promising strategy in the treatment of multiple myeloma, focusing on the tumor microenvironment and mechanisms of resistance. Therapies include monoclonal antibodies, small molecule inhibitors, and cell therapies like CAR-T cells. Numerous treatments are presently in clinical trials to assess their efficacy and safety. Various therapies, including CAR-T cells that target MSCs and PD-1/PD-L1 inhibitors, have demonstrated potential in improving immune response and decreasing minimal residual disease in relapsed or refractory patients. Bortezomib and Bone Marrow Stromal Cell Modulation have shown notable enhancements in overall survival when used in conjunction with standard treatments.

Table 5 presents a comparative analysis of various therapies, highlighting their mechanisms of action and effects on the progression of multiple myeloma. Some therapies demonstrate greater efficacy when combined, while others show promise as independent treatments. Nonetheless, further investigation is necessary to ascertain long-term efficacy and safety.

**Table 5:** Overview of MSC-targeted therapies in multiple myeloma clinical trials, highlighting mechanisms of action, trial status, outcomes, comparative effectiveness, side effects, drug class, duration of action, and targeted subpopulations

Therapy	Mechanism of Action	Clinical Trial Status	Outcomes in Multiple Myeloma	Comparative Effectiveness	Side Effects	Drug Class	Duration of Action	Targeted Subpopulation	Reference
**Anti-SLR1 Antibodies**	Blocks signaling from stromal cells in the bone marrow microenvironment	Ongoing	Shows promise in reducing tumor growth and improving response rates in combination with other treatments.	Comparatively effective in early-stage trials but needs more data for long-term efficacy.	Mild to moderate gastrointestinal disturbances.	Monoclonal Antibody	Weeks	Newly diagnosed and relapsed/refractory patients.	NCT05828511
**C-MET Inhibitors**	Targets the c-MET pathway, which is involved in the homing of myeloma cells to bone marrow	Completed	Limited efficacy as a monotherapy, but synergistic effects when combined with other treatments like proteasome inhibitors.	Less effective when used alone, more beneficial when combined.	Associated with fatigue and liver enzyme elevation.	Small Molecule Inhibitor	Short (Hours)	Relapsed/refractory myeloma patients.	[153]
**IL-6 Receptor Blockers**	Inhibits interleukin-6, a cytokine that promotes tumor growth and survival in the bone marrow	Ongoing	Demonstrated improvements in progression-free survival, especially when combined with chemotherapy.	Often more effective when combined with chemotherapy or proteasome inhibitors.	Increased risk of infections and mild neutropenia.	Monoclonal Antibody	Weeks	Patients with advanced-stage or high-risk myeloma.	[154]
**CAR-T Cells Targeting MSCs**	Modifies T-cells to target myeloma cells and MSCs in the bone marrow microenvironment	Ongoing	Early-stage trials show promise in reducing minimal residual disease and improving patient outcomes.	Higher efficacy in patients with relapsed/refractory myeloma compared to standard treatments.	Potential for cytokine release syndrome and neurotoxicity.	Cell Therapy	Months	Relapsed/refractory myeloma, particularly in patients with minimal residual disease.	NCT03548207
**Bortezomib and Bone Marrow Stromal Cell Modulation**	Inhibits proteasomal activity in both myeloma cells and bone marrow stromal cells	Completed	Significant improvement in overall survival when combined with standard myeloma therapy.	Proven highly effective when used in combination with other therapies.	Can cause peripheral neuropathy and gastrointestinal side effects.	Proteasome Inhibitor	Short (Hours)	Newly diagnosed and relapsed patients.	[155]
**Anti-Integrin Antibodies**	Targets integrins involved in myeloma cell adhesion to stromal cells	Ongoing	Early trials show reduced myeloma cell migration and reduced bone marrow microenvironment support.	Potentially effective in combination with other targeted therapies.	May cause mild infusion-related reactions.	Monoclonal Antibody	Weeks	Patients with bone marrow involvement.	[156]
**HDAC Inhibitors**	Inhibits histone deacetylases, impacting both myeloma cells and stromal cells	Ongoing	Early-phase trials indicate reduced tumor proliferation and potential synergy with other myeloma therapies.	Shows moderate effectiveness when combined with other agents but less so as a monotherapy.	Associated with fatigue, nausea, and cytopenias.	Epigenetic Modifier	Hours	Combination therapy in relapsed/refractory myeloma.	[157]
**PD-1/PD-L1 Inhibitors**	Blocks the PD-1/PD-L1 immune checkpoint pathway, promoting immune-mediated tumor cell destruction	Ongoing	Enhanced immune response and prolonged survival in combination with other immune-modulatory therapies.	Effective in combination with checkpoint inhibitors and proteasome inhibitors.	Potential for immune-related adverse events, including rash and colitis.	Immune Checkpoint Inhibitor	Weeks	Relapsed/refractory patients with immune dysregulation.	[158, 159]
**TGF-β Inhibitors**	Targets TGF-β signaling, which is involved in myeloma cell growth and resistance to chemotherapy	Ongoing	Improves myeloma cell sensitivity to chemotherapies and enhances immune responses.	Shows potential in enhancing responses to traditional chemotherapy.	Mild fatigue, skin rash, and potential for thrombocytopenia.	Monoclonal Antibody	Weeks	Relapsed/refractory myeloma with resistance to chemotherapy.	[160]
**CXCR4 Antagonists**	Blocks CXCR4 signaling, which is crucial for myeloma cell migration and homing to the bone marrow	Ongoing	Improves mobilization of myeloma cells and enhances effectiveness of other therapies.	Can significantly improve outcomes when combined with other therapies.	Bone pain, injection site reactions, and mild hematologic side effects.	Small Molecule Inhibitor	Hours	Relapsed/refractory myeloma with poor response to chemotherapy.	[161]

Within the context of MM, ncRNAs have a function in post-transcriptional gene expression regulation, exerting a significant impact on various cellular processes. The dysregulation of ncRNAs can disrupt the normal functioning of important genes that are involved in the progression of MM [162]. Moreover, they have a role in cellular development, physiology, and the pathophysiology of different human diseases, including MM [25]. Raimondi et al. have depicted that the abnormal regulation of various subclasses of ncRNAs has a significant impact on modulating important signaling pathways in the progression of MM [163]. Housekeeping ncRNAs that are expressed at high levels provide novel cancer therapies. These ncRNAs play crucial roles in the regulation of cellular activities through epigenetic, transcriptional, and post-transcriptional gene control, leading to dysregulation. Furthermore, they could modulate gene expression without modifying the DNA sequence, which made them very suitable for therapeutic interventions in diseases. Moreover, ncRNAs can function as tumor suppressors or tumor promoters and have been associated with various types of cancer [163–166].

The existence of MM cells within the BM niche modifies the activity of MSCs. In the MM disease status, after co-cultivating BM-MSCs, researchers have noticed a significant alteration in the expression of certain microRNAs (miRNAs) in BM-MSCs. These alternations ended in notable modifications to the secretory profile and osteogenic differentiation capability of the BM-MSC, which is the consequence of the MM invasion in the BM niche [14]. MiRNA is one of the other epigenetic factors that have a role in suppressing or developing MM disease. It is commonly known that miRNAs interfere with target genes, signaling molecules, and pathways within the context of the BM microenvironment, hence contributing to the pathogenesis of MM [167–169]. According to Wang et al., miR-21 was expressed in MM cells that were attached to BMSCs and was also noticeably elevated in MM cells. Furthermore, a different study showed that HMCLs had greater levels of mir-21 expression when attached to BMSCs and that blocking miR-21 considerably reduced cell viability and prevented MM cells from growing clonogenicly in stroma-free environments [170, 171]. Previous research indicates that MM-BMSCs exhibit higher expression levels of certain miRNAs, such as miR-223, miR-16, miR-519d, and miR-485-5p, than their normal counterparts [170] (Table 6).

**Table 6:** An over review of effects of epigenetic mechanisms on the interaction of mesenchymal stromal cells and MM cells

Aspect	Epigenetic Mechanisms	Impact on Mesenchymal Stromal Cells (MSCs)	Role in Myeloma (MM) Progression	Key Molecules / Factors	Effect on Chromatin and DNA Methylation	Impact on Tumor Microenvironment (TME)	Therapeutic Implications	Additional Findings	References
**Epigenetic Regulation**	Modifications to histones, DNA methylation, non-coding RNAs (miRNA, lncRNA)	Epigenetic changes regulate transcription factors, chromatin structure, and genomic stability	Epigenetic dysregulation promotes tumor growth and suppresses osteoblast differentiation	DNA methylation, histone acetylation, miRNAs, piRNAs	Changes in DNA methylation affect key genes like Runx2, suppressing osteogenesis	Epigenetic modifications drive immune evasion, cell migration, and tumor proliferation	Targeting DNA methylation and histone acetylation can reverse MM-associated bone disease and tumor progression	Histone deacetylation is critical in MM cell survival, and inhibitors like SAHA show promise for MM therapy	[126{Wu, 2023 #4650]}
**Histone Modifications**	Histone acetylation, methylation, phosphorylation, ubiquitination	Regulates chromatin accessibility, influencing gene expression	Abnormal chromatin structure promotes MM cell proliferation, immune evasion, and bone disease	Histone acetyltransferase (HAT), histone deacetylase (HDAC)	HDACs like HDAC3 suppress transcription of osteogenic genes, promoting MM progression	Aberrant HDAC activity inhibits differentiation and supports MM survival	HDAC inhibitors like vorinostat suppress tumor growth, with minimal effects on bone formation	Histone modifications like H3K9 acetylation are essential in MM progression	[172, 173]
**DNA Methylation**	Methylation of CpG islands by DNA methyltransferases (DNMTs)	Methylation regulates gene expression, impacting MSC differentiation	Aberrant methylation of key genes (e.g., HOX, Runx2) leads to bone disease and tumor proliferation in MM	DNMT3, CpG islands, G9a, CM-272	DNA hypermethylation in B-cell-specific enhancers leads to abnormal MSC function and promotes MM progression	MSCs from MM patients exhibit widespread methylation changes that impair osteogenesis	DNMT and G9a inhibitors (e.g., CM-272) prevent tumor-associated bone loss and reduce tumor burden	DNMT underexpression in MM leads to epigenetic instability, driving tumor progression	[138, 174]
**miRNA and ncRNA**	Regulation by non-coding RNAs (miRNA, lncRNA, siRNA)	ncRNAs control post-transcriptional gene expression and cellular functions in MSCs	Dysregulated miRNA expression promotes MM survival and immune evasion	miR-29b, miR-223, miR-16, miR-485-5p, lncRNA-CSR	miRNA changes alter MSC secretory profiles and osteogenesis, contributing to MM progression	miRNAs regulate key genes in the TME, influencing immune responses and tumor survival	Targeting miRNAs (e.g., miR-29b) can inhibit MM growth and improve therapy outcomes	miR-29b antagonizes HDAC4 activity, offering a therapeutic strategy in MM treatment	[162, 175]
**Role of Histone Deacetylases (HDACs)**	HDACs suppress chromatin accessibility, inhibiting gene transcription	HDAC upregulation in MM cells inhibits MSC differentiation, supporting tumor growth	Increased HDAC activity inhibits differentiation, promotes MM cell survival, and enhances bone loss	HDAC1, HDAC4, HDAC inhibitors (vorinostat, SAHA)	HDACs repress transcription of key osteogenic genes, contributing to bone disease in MM	MSCs promote MM progression through HDAC-driven immune suppression and differentiation inhibition	HDAC inhibition can enhance bone regeneration and suppress MM cell growth	HDAC4 inhibition with miR-29b mimics enhances MM cell death, providing potential for combined therapies	[176, 177]
**Runx2 Suppression**	Chromatin remodeling through HDAC and DNMT activity	Suppresses osteoblast differentiation and promotes bone disease in MM	Runx2 suppression in MSCs inhibits bone formation, supporting MM progression	NF-κB, Runx2, EZH2, G9a	Chromatin changes at Runx2 loci impair MSC function and promote MM	MSCs in the TME drive prolonged suppression of osteoblast differentiation through Runx2 repression	Inhibiting chromatin remodelers (EZH2, HDAC1) restores osteogenesis and bone health in MM patients	Runx2 suppression links epigenetic changes with impaired bone formation in MM patients	[178, 179]
**Therapeutic Potential of Epigenetic Modulation**	Targeting DNA methylation, HDAC activity, and miRNA regulation	Restoring normal chromatin and DNA methylation patterns in MSCs can reverse MM progression	Epigenetic therapies (e.g., HDAC inhibitors, DNMT inhibitors) offer potential to halt tumor growth and bone disease in MM	CM-272, SAHA, vorinostat, miR-29b mimics	Combining HDAC inhibition with miRNA targeting (e.g., miR-29b) enhances therapeutic efficacy in MM	Epigenetic therapies can restore normal MSC function, halting MM cell proliferation and restoring immune responses	Combined targeting of DNA methylation and HDACs shows potential for reversing MM bone disease	Emerging research supports combining epigenetic modulators for maximal anti-MM effects	[180{Sharbaf, 2018 #4657, 181]

### Suppress of immune checkpoints by mesenchymal stromal cells in MM

One common element of the immune system is the immunological checkpoint. Their primary function is to keep the immune response from intensifying to the point where the body’s healthy cells are harmed [182]. As the immune system’s negative regulators, immunological checkpoints can stop autoimmunity and protect tissues from being attacked by an overactive immune system [183]. When T cell proteins recognize and attach to partner proteins on other cells, including certain tumor cells, immune checkpoints are activated. Immune checkpoint proteins are the name given to these proteins [184]. When the partner proteins and checkpoint bind together, T cells get an “off” signal. This can prevent the cancer from being destroyed by the immune system, and tumor cells can exploit this to evade the immune system [185–188]. The overexpression of immunological checkpoints, which reduces effector T cell activity, is one of the immune escape routes in MM [63]. Immune checkpoints linked to MM have been identified in the past year. These include PD-1, CTLA-4, LAG-3, TIM-3, KIR, TIGIT, VISTA, and BTLA [189, 190].

The cytoplasmic domain of PD-1, also known as Programmed cell death protein 1 (CD279), is home to two tyrosine-based signaling motifs: an immune receptor tyrosine-based switch motif (ITSM) and a tyrosine-based inhibitory motif (ITIM). The molecule is a cell surface molecule with a single immunoglobulin (Ig) superfamily domain. PD-1 has two ligands: PD-L1 (B7-H1; CD274) and PD-L2 (B7-DC; CD273) [191]. As transmembrane proteins, PD-1 and its ligands have three sections: a transmembrane region, an extracellular domain, and a cytoplasmic tail [192].DCs, B cells, NKs, and activated T cells are the primary sources of PD-1 expression [193, 194]. Additionally, mast cells, DCs, and macrophages all showed reduced levels of PD-L2 expression [195]. While external stimuli have not been reported to stimulate PD-1 expression, T cell activation can induce its expression [196]. Likewise, PD-1 prevents CD4+/CD8 + T cells from penetrating the tumor, which allows myeloma cells to evade the immune system [197, 198]. When myeloma cells express PD-L1, T cell growth and function are inhibited, which results in tumor immunosuppression and an overabundance of malignant myeloma cells [189, 199].A study by Liu et al. suggests that BMSCs can inhibit the PD-1/PD-L1 pathway, which offers a novel therapy strategy for myeloma by reducing CD8 + T cell immunological response [200, 201]. in addition, Chen et al.‘s study, which examined how BMSCs affected the development of MM, showed that BMSCs increased MM cell proliferation by PD-1/PD-L1 pathway-mediated T cell immune response inhibition [202]. Therefore, one potential treatment for MM is to disrupt the PD-1/PD-L1 pathway in the BM microenvironment. In fact, many studies have been conducted on cell lines and animal models in this context and have shown that blocking the PD 1/PD L1 axis in multiple myeloma has potential therapeutic effects [203–206]. For example, in a study by Görgün et al., they investigated the effect of lenalidomide on the PD 1/PD L1 pathway and finally found that blocking this pathway induced an anti-MM immune response that could also be enhanced by lenalidomide [204]. In this regard, another study was conducted by Lesokhin and his colleagues. In this study, they investigated the effect of Nivolumab but did not show any tumor response in relapsed/refractory MM [207]. According to these results, treatment approaches involving several medications should include PD-1/PD-L1 blocking in MM.

T cell immunoglobulin and ITIM domain (TIGIT) is another immunological checkpoint linked to multiple myeloma. Recently, there has been increased focus on this lymphocyte-resident checkpoint inhibitory receptor as a major new target in cancer immunotherapy. TIGIT inhibits T cell and NK cell activity by interacting with CD155, which is expressed on antigen-presenting cells (APC) or cancer cells. This interaction may restrict both innate and adaptive immunity. According to preclinical research, TIGIT blockage may offer protection against several hematological and solid malignancies [208]. Many malignancies, including MM, express CD155 (Poliovirus receptor (PVR)). Lee et al. discovered a correlation between PVR expression and the stage of MM and a poor prognosis, indicating that PVR may serve as a prognostic marker for MM [209, 210]. While CD155 is present at very low levels on myeloma cells, it is robustly expressed on BMSCs in newly diagnosed MM patients. The interaction between NK cells and BMSC is primarily mediated by the CD115/TIGIT signaling pathway [211]. Perhaps NK cell function could be reversed by suppressing TIGIT, which could open up new therapeutic avenues. Guillerey et al., conducted a study in this field (investigating the therapeutic potential of TIGIT blockade to suppress immune responses against MM) and observed that in both mice and humans, MM progression was associated with high levels of TIGIT expression on CD8^+^ T cells. They finally concluded that blocking TIGIT with the help of monoclonal antibodies leads to better function of CD8 T cells of MM patients and suppresses MM growth. They also stated that the results obtained from this study support the development of TIGIT blocking strategies for the treatment of MM patients [212]. In addition, a phase 1/2 trial using the TIGIT blocker BMS 986,207 on multiple myeloma patients alone and in combination with pomalidomide and dexamethasone is being conducted [213, 214]. Pomalidomide is a third-generation immunomodulatory agent, whose antimyeloma mechanisms mainly involve it binding to cereblon protein, promoting the ubiquitination and degradation of IL-2 transcriptional repressors, Ikaros and Aiolos, and negatively regulating transcriptional factors IRF4 and MYC [215].

T-cells also express the inhibitory receptor known as cytotoxic T-lymphocyte-associated protein 4 (CTLA-4), which belongs to the CD28 immunoglobulin subfamily. CD80 (B7-1) and CD86 (B7-2) are its ligands [216]. They are present in APCs, including MSCs, B cells, and DCs [217]. Monocytes from the peripheral blood of humans express CD86 but not CD80. Additionally, certain human T cells can express CD80 and CD86 depending on the degree of activation [218]. When MM is active, CD4 T cell production of CTLA-4 is inappropriate and is associated with poor clinical outcomes and therapeutic outcomes. Moreover, de Nałęcz et al.‘s research showed that early relapse may be predicted by PD-1 expression in RRMM patients or lower CTLA-4 levels in NDMM patients [219]. While MSCs produce the inhibitory receptor CTLA-4, they also express multiple isoforms of the same receptor depending on the environment. In hypoxic settings, the most abundant isoform is the secreted form of CTLA-4, or sCTLA-4 [217]. BM samples from newly diagnosed MM patients showed upregulation of CTLA-4, according to a study by Braga et al. [220]. Lastly, additional research is required to determine the effectiveness of CTLA-4 inhibition as a treatment approach for malignancies, including MM (Table 7).

**Table 7:** This table relates to immunological checkpoints in multiple myeloma (MM) and their role in cancer immunotherapy

Aspect	Immune Checkpoint	Function	Role in Multiple Myeloma (MM)	Ligands/ Receptors	Mechanism in MM	Therapeutic Implications	Examples of Therapeutics	References
**Programmed Cell Death Protein 1 (PD-1)**	PD-1 (CD279)	Inhibits T cell activation	PD-1/PD-L1 overexpression prevents T cells from attacking MM cells	PD-1 ligands: PD-L1 (CD274), PD-L2 (CD273)	Inhibits CD8+/CD4 + T cells from penetrating the tumor, promoting immune evasion	Blocking PD-1/PD-L1 axis can restore T cell function and suppress tumor growth	Nivolumab, Pembrolizumab (PD-1 blockers)	[221, 222]
**T-cell Immunoglobulin and ITIM Domain (TIGIT)**	TIGIT	Inhibits T cell and NK cell activity	TIGIT/CD155 interaction in MM inhibits NK cell function, reducing immune response	TIGIT interacts with CD155 (PVR) on BMSCs and cancer cells	Suppresses innate and adaptive immunity, allowing MM progression	Blocking TIGIT can restore NK and T cell activity, suppressing MM growth	BMS 986,207 (TIGIT blocker), clinical trials ongoing	[223, 224]
**Cytotoxic T-lymphocyte-associated Protein 4 (CTLA-4)**	CTLA-4	Inhibits T cell proliferation	CTLA-4 overexpression linked to poor clinical outcomes in MM	CTLA-4 ligands: CD80 (B7-1), CD86 (B7-2)	CTLA-4 binds to CD80/CD86, suppressing T cell activation and immune responses	CTLA-4 blockade may improve immune response and MM patient outcomes	Ipilimumab, Tremelimumab (CTLA-4 inhibitors)	[216, 225]
**T-cell Immunoglobulin Mucin Domain-3 (TIM-3)**	TIM-3	Regulates T cell exhaustion	TIM-3 overexpression linked to immune evasion in MM	TIM-3 binds to Galectin-9	TIM-3/Galectin-9 binding inhibits T cell responses, promoting tumor survival	TIM-3 inhibition could improve anti-MM immune responses	TSR-022 (TIM-3 inhibitor), in clinical trials	[226]
**Lymphocyte Activation Gene 3 (LAG-3)**	LAG-3	Inhibits T cell proliferation and activation	LAG-3 overexpression impairs T cell responses, promoting immune evasion in MM	LAG-3 interacts with MHC Class II	Suppresses T cell activation, allowing MM cells to evade immune detection	LAG-3 inhibition may boost T cell function in MM patients	Relatlimab (LAG-3 blocker), U.S. FDA-approved in combination with PD-1 inhibitors	[227]
**V-domain Ig Suppressor of T Cell Activation (VISTA)**	VISTA	Suppresses T cell activation	VISTA expression on MM cells enhances immune evasion	VISTA binds to VSIG-3	Inhibits T cell proliferation and activity in the MM microenvironment	VISTA inhibitors are being explored as potential therapeutic agents in MM	CA-170 (VISTA inhibitor), in development	[228]
**B and T Lymphocyte Attenuator (BTLA)**	BTLA	Inhibits T cell activation	BTLA overexpression linked to MM progression by suppressing immune responses	BTLA interacts with HVEM (Herpesvirus Entry Mediator)	Reduces T cell activation and immune responses against MM cells	BTLA blockade may restore immune surveillance in MM	Preclinical investigations are ongoing in MM	[229, 230]
**Killer-cell Immunoglobulin-like Receptor (KIR)**	KIR	Modulates NK cell function	KIR expression modulates NK cell activity in MM, contributing to immune evasion	KIR interacts with MHC Class I molecules	Downregulates NK cell activity, allowing MM cells to escape immune detection	Blocking KIR may restore NK cell function in MM patients	Lirilumab (KIR blocker), ongoing trials in MM	[231]
**Combination Therapy**	PD-1/CTLA-4 and other checkpoints	Enhances immune response	Combining PD-1 and CTLA-4 inhibitors shows enhanced anti-MM activity	CTLA-4 + PD-1 inhibitors	Synergistic effect on immune response, activating both T and NK cells against MM	Combination therapies are key for durable responses and long-term survival	PD-1 + CTLA-4 combinatorial trials show improved outcomes in cancer	[232]

### Mesenchymal stromal cells induce drug resistance in MM

MM, plasma cell malignancy, remains incurable until now, so anti-myeloma drugs should improve its prognosis. Despite progress in disease treatment, resistance to therapy and relapse has occurred [233]. There are two categories of drug resistance: intrinsic and extrinsic mechanisms. The intrinsic process consists of modifications to the drug target, overexpression of drug efflux pumps, genetic and epigenetic changes, and dysregulation of intracellular signaling cascades, including those that govern autophagy, DNA repair, and apoptosis. Extrinsic mechanisms take place in the BM niche, where endothelial cells, fibroblasts, osteoblasts, osteoclasts, and immune cells are closely connected to each other as well as the TME [234]. The interactions between the BM microenvironment, cell adhesion molecules to the extracellular matrix (ECM), and other components of the TME release soluble factors like insulin-like growth factor (IGF)-1 and IL-6, as well as cell cycle inhibitors, anti-apoptotic Bcl-2 family members, and ABC drug transporters in the malignant cell. These interactions also activate pathways related to signal transduction, hypoxia, autophagy, angiogenesis, metabolism reprogramming, and apoptosis resistance, all of which culminate in drug resistance [235–237]. Demonstrated that Hypoxia induces stromal cells to secrete extracellular vesicles with increased miR-140–5p and miR-28–3p that are transferred to multiple myeloma cells and drive drug resistance by increasing the MAPK signaling. These mirRNAs target SPRED1 to confer drug resistance in multiple myeloma [238].

There are 49 types of ABC transporters with 7 subfamilies based on their sequence into groups ABCA to ABCG. Because of the importance of BM in cell growth and its expansion in the progression of MM, researchers have depicted that myeloma cells interact with BMSCs and the ECM, facilitating their growth and better survival and making them resistant to drugs [239]. BMSCs release cytokines and chemokines that can bind to both PC and malignant PC. They also produce immunoglobulin, which stimulates high levels of immunoglobulins and activates the unfolded protein-stress response (UPR) and endoplasmic reticulum (ER) pathways. Additionally, they activate the nuclear factor kappa B (NF-κB), phosphoinositide 3 kinase (PI3K)/Akt, and mitogen-activated protein kinase (MAPK) signaling pathways, which in turn suppresses caspase cleavage and apoptosis in myeloma cells caused by chemotherapy [233, 239].

Some miRNAs were identified as having a role in drug resistance. For instance, the upregulation of miR-21 hasbeen linked with doxorubicin and dexamethasone resistance in MM cells.Bound of BMSCs to myeloma cellsupregulates expression of miR-21 [240].

Co-culture of BMSCs and MM cells increases levels of IL-6 that lead to downregulation of miR-15a, These interactions enhanced the protection of MM cells from apoptosis induced by melphalan and bortezomib [241].

As a result of exposure to a single chemotherapeutic agent, cancer cells may develop multidrug resistance (MDR), a kind of ABC transporter, which makes them resistant to a variety of structurally and functionally unrelated medications. The overexpression of resistance proteins, such as P-glycoprotein (P-gp), which are members of the ATP class of drug transporters, is one of the most significant mechanisms in MDR tumors. These are transporters of plasma membrane drugs that control the extrication of chemotherapeutic agents from the plasma membrane of cancer cells.In many malignancies, an elevated level of P-gp expression is also linked to a poor prognosis and a poor response to chemotherapy. In addition, P-gp expression rises in patients by up to 75% after therapy. However, there is evidence that more recent forms of treatment, such as proteasome inhibitors and immunomodulatory medications, are similarly hampered since they are P-gp substrates. Similarly, numerous drugs often employed in combination treatment for MM are likewise classified as P-gp substrates [242–245].

HMG1, a nonhistone protein located in the nucleus that is essential for transcription, damage repair, DNA organization, and replication, is another element that affects regenerative chemotherapy. When HMGB1 is overexpressed in comparison to normal tissues, it can lead to several types of tumors developing and progressing, such as invasion, metastasis, inflammation, and angiogenesis [246–248]. According to Liu et al.‘s study, the use of HMGB1-neutralizing antibodies makes cells more susceptible to chemotherapy and increases the release of HMGB1 from leukemia cells. According to an in vivo investigation, HMGB1 can increase lung cancer cells’ resistance to drugs, which in turn can lead to tumor growth [249]. The expression of HMGB1, which was increased in MM cells, was negatively linked with the lifetime of MM patients. Bortezomib-resistant MM cells also exhibited elevated HMGB1 expression, and MM cells responded better to treatment in vivo when HMGB1 levels were lowered [250].

### Mesenchymal stromal cells and the senescence of MM

Cellular senescence is a process marked by the permanent halt of cell division and has significant implications in cancer biology, particularly in relation to MM. While this mechanism acts as a powerful tumor suppressor by stopping damaged or stressed cells from multiplying uncontrollably and potentially developing mutations that lead to cancer, it can also paradoxically promote cancer progression in specific situations. As such, senescence represents a complex factor with both beneficial and detrimental effects within the context of malignant conditions [251]. Senescence plays a crucial role in suppressing tumors by stopping the division of cells that may become cancerous. This process serves as a protective barrier during the initial phases of cancer, when cells undergo mutations with the potential to develop into malignancies. By preventing these altered cells from proliferating and spreading, senescence effectively hinders tumor formation and advancement [252–254].

Despite its tumor-suppressive role, senescence can also facilitate cancer progression through the senescence-associated secretory phenotype (SASP). Senescent cells, even though they do not proliferate, continue to be metabolically active and can impact their surroundings by releasing various inflammatory cytokines, growth factors, and proteases. These released substances can negatively affect the nearby tissue by promoting inflammation, disturbing the structure of the tissue, and encouraging the growth of neighboring pre-cancerous or cancerous cells. Furthermore, in an aging body, the buildup of senescent cells could worsen these effects and potentially create an environment that supports cancer progression [255, 256].

In the context of MM, senescence is involved in various aspects of disease development, immune imbalance, and tumor characteristics. Senescence symptoms, such as a reduction in the capacity to proliferate and modifications in the secretion of pro-inflammatory cytokines, are observed in MM cells. Additionally, MM is linked to immunometabolic irregularities associated with immunosenescence, leading to advanced aging and an inflammatory immune reaction known as inflammaging, which influences disease advancement [257, 258].

Senescence is induced in response to various cellular stressors, including oncogenic mutations. In MM, the activation of cellular senescence significantly impacts tumorigenesis by governing cellular responses to stress and oncogenic signals [259]. Notably, MM cells possess the capability to change their senescence phenotype, which in turn impacts their behavior as well as the TME. This modification plays a significant role in the progression of the disease and its resistance to therapeutic interventions [260, 261].

Mesenchymal stromal cells have been found to play a vital part in the aging and advancement of MM by interacting with the TME. These versatile precursor cells contribute significantly to regulating the TME. Importantly, their behavior can be influenced by MM cells, resulting in either supportive or inhibitory effects on tumor development [14, 262]. MSCs are recognized as a support system for myeloma cells and can be activated by myeloma cells to produce autotaxin, which plays a significant role in lysophosphatidic acid (LPA) biosynthesis. This signaling pathway involves LPA1 and LPA3 receptors which determine the destiny of MSCs - with silenced LPA3 MSCs displaying age-related characteristics while promoting MM progression and angiogenesis related to tumors. Conversely, silenced LPA1 MSCs show resistance against senescence and have demonstrated the potential to slow down MM progression [263, 264].

The senescence of mesenchymal stromal cells in MM is a multifaceted process involving intricate changes in gene expression, extensive modulation of signaling pathways, and substantial influence from the TME on MSC behavior. The expression levels of senescence-related genes in MSCs are closely linked with MM prognosis and are implicated in conferring resistance to proteasome inhibitors through their impact on lipid metabolism [265]. Additionally, the mechanism of aging that impacts the BM-derived mesenchymal stromal cells (BM-MSCs) seems to be closely linked with the progression of MM. A set of genes that regulate steroid biosynthesis, the cell cycle, and metabolism play a significant role in determining the prognosis of MM and the immune status of susceptible groups [265, 266]. Notably, the differentially expressed genes observed in both senescent MSCs and MM tumor cells hold significant relevance for understanding the disease’s pathogenesis, influencing not only its prognosis but also impacting the immune status within high-risk groups.

Some studies suggested that the PI3K-AKT-mTOR signaling pathway is a crucial mediator in the dynamic interplay between MM cells and MSCs. Notably, MSCs derived from MM patients with active disease exhibit a distinct gene expression profile characterized by an evident overrepresentation of the PI3K-AKT-mTOR hallmark gene set. Specifically targeting this intricate signaling cascade within MSCs using a pan-PI3K inhibitor (like pictilisib) has been shown to selectively impede the proliferation of MM-associated MSCs while simultaneously diminishing their supportive role in promoting tumor progression [267]. Concurrently, the NF-κB signaling pathway plays a pivotal role in the pathophysiology of MM, significantly impacting the survival, proliferation, and chemoresistance of malignant PC. It is triggered by mutations linked to cancer, by the autocrine and paracrine release of growth factors and cytokines, or by direct contact with different elements found in the microenvironment of the BM. It’s interesting to note that NF-κB transcription factors also mastermind important modifications to MSC functional properties that promote the progression of cancer. They play a crucial role as effectors or regulators of pathways that control interactions between MM cells and MSCs on several levels [104].

The behavior of BM-MSCs is greatly influenced by MM cells, which results in modifications to their gene expression profile, capacity to generate bone tissue, rate of cell division, and levels of senescence indicators. This two-way complex interplay involves not only the secretion of soluble factors but also EVs that transport various molecules capable of shaping the BM microenvironment to promote disease progression. MSCs derived from MM patients demonstrate enhanced motility and pro-tumor activity, thought to be driven by IRE1a-induced phosphorylation of FilaminA (FLNA), which in turn enhances their migration. Disrupting this IRE1a-FLNA axis has the potential to interfere with MSC response to MM stimuli and potentially mitigate cell-cell adhesion-mediated drug resistance [268].

The response of MSCs to novel MM drugs like melflufen is another crucial topic of recent research, as these drugs can significantly impact the proliferation and differentiation of MSCs, which play a pivotal role in the pathogenesis of MM. Studies have demonstrated that melflufen exerts cytotoxic effects on BM-MSCs and interferes with their potential to differentiate into adipocytes and osteoblasts, as well as their involvement in angiogenesis [269]. In addition, investigations demonstrate the importance of exosomal miR-483-5p produced from BM-MSCs in stimulating the tumor suppressor gene TIMP2 to advance the malignant development of MM [270]. These findings underscore the essential role played by BMSC-derived exosomal miRNAs in driving MM progression, indicating their potential utility as diagnostic markers and therapeutic targets.

In general, it can be said that in the context of MM, senescence is known to have a multifaceted role, acting as both a tumor-suppressive mechanism and contributing to disease progression. The regulation of senescence and the formation of disease pathogenesis are significantly influenced by the interactions that occur between mesenchymal stromal cells and MM cells in the TME. Delving into the molecular mechanisms driving MSC senescence in MM, including changes in gene expression and signaling pathways, is crucial for devising targeted therapeutic approaches to improve patient outcomes.

### Reprogramming of mesenchymal stromal cells in MM

Many strategies for treating MM bone disease have been discovered recently, particularly in relation to the utilization of MSCs [271]. One of the uses of MSCs is to use them to produce iPSC [272, 273]. Studies have shown that MSCs are more efficient in reprogramming compared to other somatic cells [274, 275]. Osteoblasts, which are derived from MSCs, are in charge of the development of new bone. According to reports, myeloma cells prevent MSCs from differentiating into mature osteoblasts [42, 276, 277]. In fact, lineage plasticity is exhibited by osteoblasts and adipocytes, which share a common progenitor that is produced from MSCs [278]. One feature of MM is the suppression of bone formation. MSCs can develop into adipocytes, but myeloma cells prevent them from doing so [279]. BM adipocytes are now understood to be more than merely inert “filler cells”; they are significant regulators of bone remodeling [280, 281]. According to Liu et al.‘s study, myeloma cells have the capacity to reprogram normal BM adipocytes, giving them the capacity to resorb bone in myeloma patients who are in remission [282, 283]. Lemaitre et al. conducted a study with the aim of determining whether MM-MSCs also contribute to disease recurrence. Ultimately, they discovered that changes, possibly brought about by epigenetic reprogramming, caused by the presence of MM PC in the BM caused the MSC to change into a persistent pro-tumoral phenotype. Furthermore, they proposed that MSC can contribute to the recurrence of MM by encouraging the development of minimal residual cells [284]. Recent studieshave shown that myeloma-induced bone disease is influenced by bone marrow adipocytes (BMAds) and that BMAds that have undergone MM-reprogramming are a part of this process. MM-MSCs also exhibit decreased osteogenic potential and changes in the expression of transcripts related to the pathophysiology of MM illness (IL-6). There is evidence that MM-MSCs have abnormal secretory profiles and senescent traits that might hinder bone production [221, 285, 286].

After the MM niche has stabilized, when stromal, endothelial, or osteolineage cells come into direct touch with malignant PC, the immediate BM microenvironment is reprogrammed, either leading to the facilitation of immune escape and the activation of cytokines that promote MM cells [287, 288]. These reactions provide MM cells additional signals for growth, and as a result, a favorable TME is generated as they progressively become independent of the initial support of their typical habitat. Notably, MM-MSCs have different gene profiles from their counterparts in normal MSCs [289, 290]. Moreover, as previously indicated, MM cells may now function remotely to create new premetastatic niches for tumor spread in other bone areas by secreting growth factors, cytokines, and exosomes that alter the ECM in these new locations [291].

The multiline age differentiation potential of BMSCs is contingent upon their growth status and their interactions with their microenvironment in both healthy and diseased states. This is because BMSCs are pluripotent progenitor cells that possess the ability to self-renew and the potential to differentiate into multiple mesoderm lineage cells, such as chondrocytes, adipocytes, and osteoblasts [292–294]. For example, normal epigenetic reprogramming and differentiation capabilities of BMSCs into functional osteoblasts are disrupted throughout aging and in a variety of inflammatory and malignant situations, leading to increased adipogenesis and decreased osteogenesis [295]. Since The majority of people with MM are old, with a typical diagnosis age of about 70 years, The remarkable parallels between the alterations seen in BMSCs from elderly persons and those found in BMSCs from pathologic inflammatory diseases affecting the BM should not be overlooked [296].

### Ethical and therapeutic challenges of mesenchymal stromal Cell-Based therapies in multiple myeloma

The therapeutic manipulation of mesenchymal stromal cells (MSCs) in clinical applications for patients with multiple myeloma (MM) presents notable ethical challenges, especially regarding the potential risks linked to tumor promotion. Mesenchymal stem cells (MSCs) are the subject of extensive research due to their regenerative and immunomodulatory properties, with applications in tissue repair and immune modulation. Multiple studies have shown that MSCs may unintentionally facilitate tumor progression, especially in hematologic malignancies such as MM, where the bone marrow microenvironment is already affected by cancerous plasma cells. The interactions between mesenchymal stem cells and malignant plasma cells in multiple myeloma can create a more supportive tumor microenvironment, potentially complicating treatment outcomes and disease prognosis [297].

### Mechanisms of tumour promotion by MSCs

Mesenchymal stem cells (MSCs) can promote tumor growth through mechanisms including angiogenesis, immune evasion, and cellular differentiation, thereby enhancing the survival and proliferation of malignant cells in the bone marrow niche. Research indicates that exosomes derived from mesenchymal stem cells (MSCs) and cytokines including IL-6, VEGF, and TGF-β are critical in tumor proliferation, immune suppression, and resistance to chemotherapy.IL-6 secreted by MSCs functions as a growth factor for MM cells, enhancing their survival and augmenting resistance to apoptosis. Moreover, MSCs have demonstrated the ability to augment the stem-like characteristics of MM cells, thereby promoting their dormancy and potential for relapse following treatment. These interactions highlight the need for additional research into the role of MSC-derived signals in the intricate biology of MM. In multiple myeloma, mesenchymal stem cells demonstrate a modified gene expression profile relative to healthy cells, characterized by notable upregulation of genes linked to tumor-supportive pathways, such as NF-kB, STAT3, and PI3K/AKT signaling. The pathways enhance tumor cell proliferation and contribute to the immune-privileged environment of the bone marrow, enabling malignant plasma cells to evade immune surveillance. Moreover, mesenchymal stem cells in the multiple myeloma microenvironment may undergo epigenetic modifications that promote tumor growth and contribute to drug resistance. The findings highlight the dual role of MSCs in regenerative medicine and cancer progression, indicating the need for a cautious approach in their therapeutic application [298, 299].

### Ethical considerations in MSC therapies

The ethical considerations surrounding MSC therapies include the risk of tumor promotion and substantial concerns regarding patient safety, especially in instances where MSCs are genetically modified for therapeutic purposes. Genetically modified mesenchymal stem cells (MSCs) offer potential advantages for tissue regeneration and therapeutic gene delivery; however, they also present significant risks, particularly in patients with pre-existing malignancies like multiple myeloma (MM).

Studies indicate that genetic modifications intended to enhance the regenerative potential of mesenchymal stem cells (MSCs), such as the overexpression of fibroblast growth factor-2 (FGF-2) or hepatocyte growth factor (HGF), may unintentionally promote tumor growth. A recent meta-analysis of MSC therapies in oncology revealed that modified MSCs may develop tumorigenic properties under prolonged culture conditions, especially with extensive passaging or exposure to pro-inflammatory cytokines. The potential for horizontal gene transfer between modified MSCs and malignant cells is a significant concern, underscoring the necessity for comprehensive safety evaluations prior to clinical application. Studies indicate that MSCs derived from MM patients possess genetic mutations that increase their susceptibility to malignant transformation. This raises important concerns regarding their application in autologous transplantation contexts, where the reinfusion of modified MSCs may exacerbate tumor progression. Recent investigations have demonstrated that MSCs possess significant plasticity, enabling them to adapt dynamically to their microenvironment. The adaptability of MSCs, although beneficial in regenerative medicine, may elevate the risk of these cells unintentionally developing tumorigenic characteristics in the context of malignancy [300, 301].

### Risk mitigation strategies and regulatory considerations

Considering these risks, thorough preclinical testing is crucial for assessing the long-term effects of MSC therapies, especially in oncological contexts. Regulatory agencies, including the European Medicines Agency (EMA) and the U.S. Food and Drug Administration (FDA), underscore the importance of rigorous safety evaluations, especially regarding MSC-based treatments in cancer patients. This regulation critically involves comprehensive analyses of genomic stability to ensure that MSCs do not acquire harmful mutations during ex vivo expansion. High-throughput sequencing and advanced transcriptomic profiling are increasingly utilized to evaluate the stability of MSCs prior to therapeutic application. Moreover, risk stratification models are being created to classify patients according to their probability of encountering adverse effects from MSC therapies. The longitudinal tracking of MSC-treated patients through imaging and biochemical markers is essential for the early detection of tumor development. MRI, PET-CT scans, and liquid biopsy techniques are under investigation as potential methods for monitoring the biodistribution and behavior of infused MSCs over time. Furthermore, computational modeling of MSC-tumor interactions is becoming a valuable method for predicting potential oncogenic outcomes prior to clinical application. Integrating machine learning algorithms with biological data allows researchers to enhance predictive models for patient selection and MSC manipulation strategies. Ethical oversight and strict guidelines for informed consent are essential for ensuring that patients comprehend the potential oncogenic risks linked to MSC therapies. Due to the complexities of MSC interactions in tumor microenvironments, ethical committees are promoting increased transparency in patient education materials. Several institutions are contemplating the formation of independent monitoring boards to assess long-term outcomes of MSC treatments. The objective of these efforts is to reconcile the prospective advantages of MSC-based regenerative medicine with the imperatives of patient safety and ethical integrity [302, 303].

### Therapeutic implications

The application of mesenchymal stem cells (MSCs) in the treatment of multiple myeloma presents potential advantages, accompanied by considerable ethical issues. Mesenchymal stem cells (MSCs) possess the ability to promote tissue regeneration and modulate immune responses, making them a promising candidate for innovative therapeutic strategies. However, their capacity to facilitate tumor progression necessitates thorough examination. Recent evidence highlights the importance of understanding the tumor-promoting mechanisms of MSCs to mitigate risks while harnessing their therapeutic benefits. Wang et al. [304]. conducted a study indicating that bone marrow mesenchymal stem cells (BM MSCs) in multiple myeloma (MM) contribute to drug resistance and disease relapse. A sub-population of MSCs, termed inflammatory MSCs (iMSCs), has been identified as specific to the MM bone marrow microenvironment and is associated with drug resistance. Analysis of public expression data from unexpanded BM MSCs revealed a positive correlation between iMSC signature expression and minimal residual disease.

Research by Zhang et al. [238]. demonstrated that hypoxia enhances the release of small extracellular vesicles (sEVs) from bone marrow stromal cells (BMSCs). Small extracellular vesicles (sEVs) exhibited a greater attenuation of bortezomib sensitivity in multiple myeloma cells compared to those derived from bone marrow stromal cells (BMSCs) under normoxic conditions. RNA sequencing indicated increased levels of miR-140-5p and miR-28-3p in hypoxic BMSC-derived sEVs, which imparted bortezomib resistance in multiple myeloma cells through the targeting of SPRED1, a regulator of MAPK activation. Studies on MSC-derived extracellular vesicles (EVs) suggest a potential approach to reduce tumor-promoting properties while maintaining regenerative benefits. The findings indicate that while MSCs possess therapeutic potential, their clinical application in MM requires thorough optimization and risk assessment. The development of MSC-based therapies requires a careful balance between innovation and patient safety, emphasizing the importance of ethical considerations in therapeutic advancement. Strengthening ethical frameworks is crucial to ensure that MSC-based interventions do not inadvertently compromise patient safety. Transparency in clinical research, thorough informed consent processes, and regulatory oversight are critical for mitigating the risks associated with MSC-based therapies. The integration of these safeguards allows for the advancement of MSC therapeutics while reducing the risk of adverse oncogenic outcomes.

### Future perspectives and open key questions

Future perspectives in MSC-based therapeutic strategies highlight a promising yet complex avenue for advancing multiple myeloma (MM) treatment. The detailed table included in this work provides a framework for understanding emerging therapies and their potential clinical applications by categorizing them based on mechanisms of action, MSC-specific impacts, delivery methods, and challenges. For instance, epigenetic inhibitors and immune checkpoint blockade are not only highlighted as therapeutic strategies but also contextualized within their capacity to reprogram MSCs or alleviate their tumor-supportive roles. The table underscores the necessity of tailoring these approaches to individual patient characteristics, such as genetic profiles and disease subtypes, to enhance treatment efficacy. Combinatorial approaches, such as integrating MSC modulation with standard MM treatments like chemotherapy, immunotherapy, and bone marrow transplantation, are also addressed as key strategies. These perspectives demonstrate that a multi-faceted approach targeting MSCs could disrupt angiogenesis, inhibit MM cell growth, and mitigate bone disease, providing a more robust therapeutic framework. Additionally, the table provides critical insights into using MSCs as diagnostic tools and therapeutic vehicles, highlighting their dual utility in monitoring disease progression and delivering targeted treatments. For example, MSC-derived exosomes are shown as a novel and promising modality due to their immunomodulatory properties and capacity for transferring therapeutic molecules. However, significant challenges remain, as detailed in both the text and table, such as addressing the heterogeneity of MSC populations, long-term safety, and the ethical considerations of MSC-based therapies. By illustrating how these therapies can be optimized and integrated, the table complements the discussion of future directions and highlights the pressing questions that need to be resolved for clinical translation. This alignment between text and tabular content provides a cohesive roadmap for researchers to explore the therapeutic potential of MSCs in MM further (Table 8).

**Table 8:** Detailed comparison of emerging therapies targeting mesenchymal stromal cells (MSCs) in multiple myeloma (MM), including mechanisms of action, MSC-specific impacts, delivery methods, biomarkers, MSC-specific modifications, challenges, combination strategies, clinical trial statuses, and safety concerns

Therapy	Mechanism of Action	Impact on MSC Modulation	Therapeutic Examples	Delivery Methods	Targeted Biomarkers	MSC-Specific Modifications	Challenges	Potential Combination Strategies	Clinical Trial Status	Safety Concerns	References
**Epigenetic Inhibitors**	Target DNA methylation, histone acetylation, or chromatin remodeling.	Alter MSC gene expression, reducing tumor-supportive interactions.	DNMT inhibitors (e.g., decitabine), HDAC inhibitors (e.g., panobinostat).	Oral or IV infusion.	Histone deacetylases, DNMT1.	Reprogram MSC gene expression to reduce tumor signaling.	Off-target effects, limited MSC specificity.	Combine with immune checkpoint inhibitors to enhance anti-tumor effects.	Phase I/II trials ongoing	Systemic toxicity due to epigenetic alterations.	[305]
**Immune Checkpoint Blockade**	Block immune checkpoint pathways (e.g., PD-1/PD-L1, CTLA-4).	Reduce MSC-induced immune suppression, boosting T-cell-mediated anti-tumor responses.	Anti-PD-1 (e.g., nivolumab), Anti-PD-L1 (e.g., atezolizumab).	IV infusion.	PD-1, CTLA-4.	Reduce MSC-mediated immunosuppressive cytokines.	Risk of immune-related adverse events (irAEs).	Pair with epigenetic inhibitors to restore immune activity.	Approved for other cancers; trials in MM ongoing	Risk of autoimmune disorders.	[232, 306]
**CAR-T Therapy**	Engineer T-cells to target specific antigens on cancer cells.	MSCs can be reprogrammed to secrete supportive cytokines for CAR-T function.	BCMA-directed CAR-T therapies (e.g., idecabtagene vicleucel).	Cell therapy, personalized.	BCMA.	Enhance MSC cytokine production for CAR-T persistence.	MSCs may promote immune escape and resistance.	Use with MSC-modulating agents to improve CAR-T persistence and efficacy.	Approved for MM	Cytokine release syndrome (CRS), neurotoxicity.	[307]
**Small Molecule Inhibitors**	Target signaling pathways critical to MSC and tumor crosstalk (e.g., CXCR4, VEGF, Wnt).	Disrupt MSC-mediated tumor survival and angiogenesis.	CXCR4 inhibitors (e.g., plerixafor), VEGF inhibitors (e.g., bevacizumab).	Oral or subcutaneous injection.	CXCR4, VEGF.	Modify MSC signaling to inhibit angiogenesis.	Potential toxicity and off-target effects.	Combine with immune-based therapies to enhance anti-tumor responses.	Plerixafor approved; others in trials	Tumor resistance due to redundancy in signaling pathways.	[308]
**MSC-Derived Exosome Therapy**	Use MSC-derived exosomes engineered for therapeutic effects.	Deliver anti-tumor molecules or modulate MSC communication with the tumor microenvironment.	Engineered exosomes loaded with siRNA or small molecules.	IV infusion or direct tumor injection.	MSC surface markers (e.g., CD44, CD73).	Load exosomes with anti-MM molecules or siRNA.	Scalability, delivery specificity to tumor niche.	Pair with immune checkpoint blockade to reduce immune suppression.	Preclinical studies	Potential for off-target effects in healthy tissues.	[309]
**Anti-Myeloma Antibodies**	Target tumor-specific antigens to mediate cell death.	May indirectly modulate MSC behavior by reducing tumor-driven signals.	Anti-CD38 (e.g., daratumumab), Anti-SLAMF7 (e.g., elotuzumab).	IV infusion.	CD38, SLAMF7.	Decrease MSC-tumor cell adhesion.	Resistance due to MSC-mediated immune evasion.	Combine with MSC-targeting therapies for synergistic effects.	Approved for MM	Infusion-related reactions, cytopenias.	[310, 311]
**Proteasome Inhibitors**	Inhibit proteasome activity, leading to cancer cell apoptosis.	Affect MSCs by altering cytokine secretion and reducing support for MM cells.	Bortezomib, carfilzomib.	IV or subcutaneous injection.	Proteasome subunits (e.g., β5).	Suppress MSC secretion of pro-tumor cytokines.	Development of drug resistance, side effects.	Combine with immunomodulatory drugs for enhanced efficacy.	Approved for MM	Peripheral neuropathy, GI toxicity.	[312]
**Immunomodulatory Drugs (IMiDs)**	Modulate the immune system and microenvironment to target MM cells.	Influence MSCs by altering their cytokine profiles and reducing tumor support.	Lenalidomide, pomalidomide.	Oral.	Cereblon.	Enhance anti-tumor immunity and MSC immunomodulation.	Risk of thromboembolic events, cytopenias.	Use with dexamethasone and proteasome inhibitors for synergistic effects.	Approved for MM	Risk of venous thromboembolism.	[313]
**Bisphosphonates**	Inhibit osteoclast-mediated bone resorption.	Affect MSC differentiation, promoting osteoblast activity and reducing MM-induced bone disease.	Zoledronic acid, pamidronate.	IV infusion.	Bone remodeling enzymes.	Promote MSC osteoblastic differentiation.	Potential renal toxicity, osteonecrosis of the jaw.	Combine with anti-myeloma therapies to manage bone disease.	Approved for MM-related bone disease	Renal impairment, jaw osteonecrosis.	[314, 315]

## Conclusion

Extensive research highlights the critical importance of BM MSCs in the development and progression of MM. These cells play essential roles in processes including drug resistance, tumour growth, homing, survival, and immune modulation. The immune microenvironment, influenced by bone marrow mesenchymal stem cells, encompasses pro-inflammatory signalling, immune checkpoints, and immune suppression, which collectively promote multiple myeloma progression. The interaction between MM cells and BM MSCs occurs through direct cell-to-cell contact and soluble cytokines, resulting in significant changes in MSCs. The changes include epigenetic modifications like DNA methylation and histone modifications, dysregulation of non-coding RNAs, induction of senescence, altered gene expression, and reprogramming into MM-supportive MM-MSCs. The pathological alterations in BM MSCs are closely associated with the advancement of MM, underscoring their essential role in the disease’s pathology. The elucidation of molecular mechanisms governing bidirectional communication between multiple myeloma cells and bone marrow mesenchymal stem cells has yielded important insights into the pathogenesis of multiple myeloma. The findings elucidate critical pathways that can be targeted to disrupt multiple myeloma cell survival and proliferation within the bone marrow microenvironment, thereby facilitating the development of innovative, targeted therapies to impede disease progression. Despite notable advancements, numerous critical research areas continue to be insufficiently investigated. Future research should focus on elucidating the heterogeneity of mesenchymal stem cells within the bone marrow niche, as this diversity is likely significant in multiple myeloma progression and therapeutic resistance. Advanced technologies like single-cell RNA sequencing (scRNA-seq) and spatial transcriptomics have significant potential for mapping the cellular landscape of the bone marrow microenvironment. These techniques elucidate the transcriptional and spatial variations among MSC subsets, enhancing the comprehension of their functions in MM. Single-cell analyses may identify rare MSC populations that either promote or inhibit MM progression. Additionally, spatial transcriptomics could elucidate the interactions between MSCs, MM cells, and other niche components in specific regions of the BM. Longitudinal studies that integrate these technologies with epigenetic profiling may yield insights into the temporal evolution of MSC alterations throughout the progression and treatment of MM. Tracking changes in DNA methylation and histone modifications over time may elucidate the transformation of normal HD-MSCs into MM-supportive MM-MSCs. Exploring the role of non-coding RNAs and extracellular vesicles in mediating communication between multiple myeloma and mesenchymal stem cells represents a promising research avenue. Analyzing the contributions of these factors to tumour proliferation, immune evasion, and therapy resistance may reveal new therapeutic targets. Investigating the metabolic reprogramming of MSCs in the MM microenvironment may elucidate how metabolic alterations facilitate MM cell survival and contribute to the suppression of immune responses. The utilization of in vitro organoid models alongside in vivo imaging technologies is essential for investigating the dynamic interactions between MM cells and MSCs in a controlled environment. These models facilitate the assessment of the efficacy of potential therapeutic interventions aimed at the bone marrow niche with enhanced precision. In conclusion, the application of these findings in clinical practice necessitates comprehensive preclinical studies to assess the impact of targeting MSC-MC interactions on MM progression, bone disease, and drug resistance. Efforts must prioritize the development of combination therapies that concurrently target BM-MSCs, immune suppression, and MM cells to enhance treatment efficacy and reduce relapse rates.

## Electronic supplementary material

Below is the link to the electronic supplementary material.

Supplementary Material 1

## Data Availability

No datasets were generated or analysed during the current study.
