# Supplementary figures and images for: Mesenchymal stromal cells in bone marrow niche of patients with multiple myeloma: a double-edged sword
Source: Cancer Cell Int. 2025 Mar 26;25:117. doi: 10.1186/s12935-025-03741-x (PMC11948648; doi:10.1186/s12935-025-03741-x)

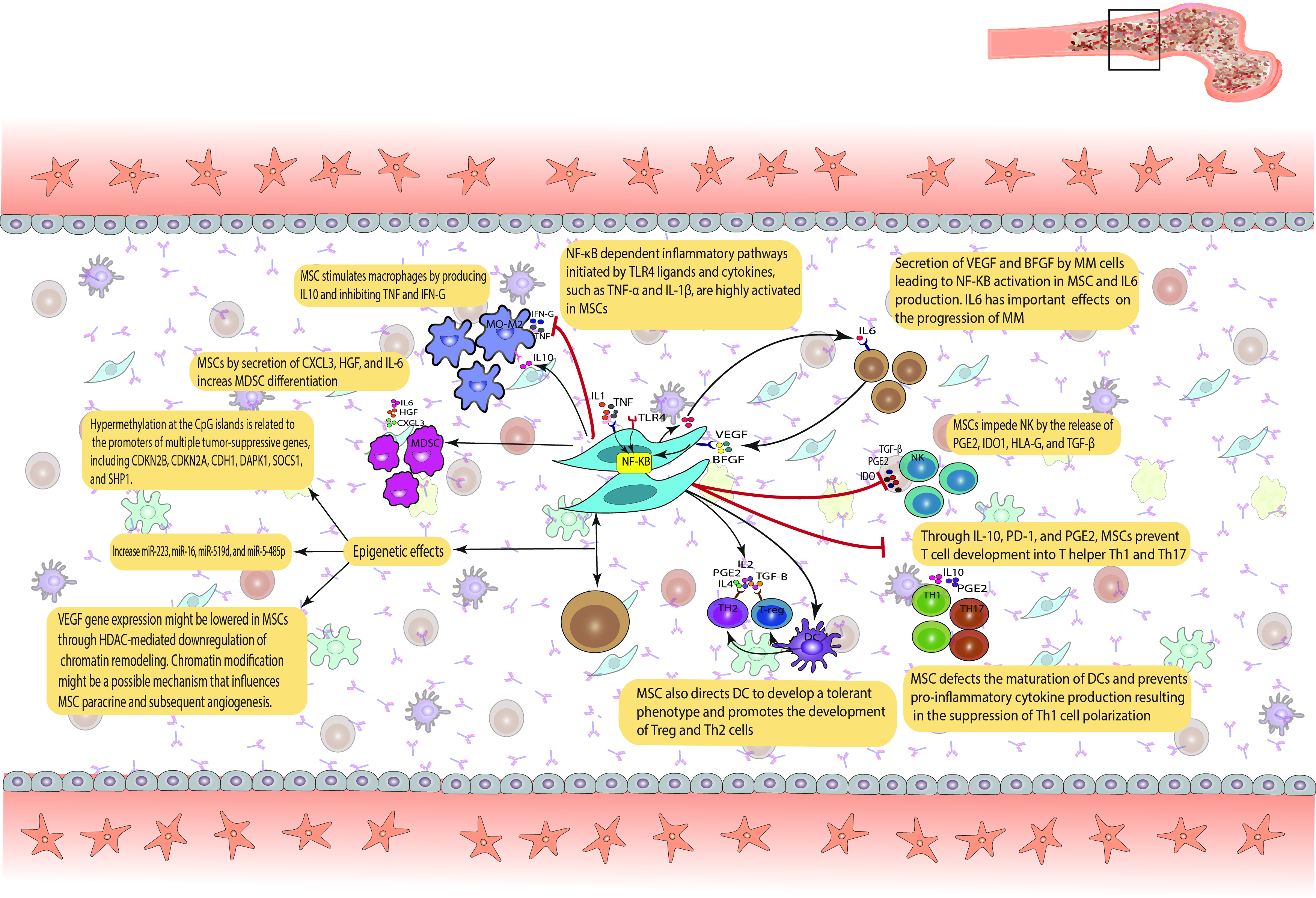

Supplement: Supplementary file 1 — Supplementary Material 1 [file 12935_2025_3741_MOESM1_ESM.jpg]
